# Temporal Dynamics of Influenza-Associated Anxiety Symptom Linguistic Markers on Weibo (2023-2024): Observational Study

**DOI:** 10.2196/88849

**Published:** 2026-08-07

**Authors:** Yifan Ou, Gert-Jan de Bruijn, Peter Johannes Schulz

**Affiliations:** 1 Department of Communication Science, University of Antwerp, Sint-Jacobstraat 2, Antwerp, 2000, Belgium; 2 School of Social Sciences, University of Manchester, Manchester, England, United Kingdom; 3 Faculty of Communication, Culture, and Society, Università della Svizzera italiana, Lugano, Switzerland; 4 Wee Kim Wee School of Communication and Information, Nanyang Technological University, Singapore, Singapore; 5 Department of Communication & Media, Ewha Womans University, Seoul, Republic of Korea

**Keywords:** influenza season, Weibo, anxiety symptoms, linguistic marker, affective dynamics

## Abstract

**Background:**

Influenza seasons may be associated with increased anxiety-related expressions on social media. Social media can reflect population-level emotional expression patterns in real time.

**Objective:**

The aim of the study is to characterize diurnal and full-season dynamics of anxiety-related language during the 2023‐2024 influenza season in China and its association with influenza activity.

**Methods:**

We retrieved Sina Weibo posts in February 2025 covering September 4, 2023, to April 28, 2024. Posts containing *Diagnostic and Statistical Manual of Mental Disorders* (*DSM*)–based anxiety terms were cleaned and deduplicated (N=169,728 → 106,440). We first linked weekly influenza incidence with anxiety-related postings. Then, we plotted diurnal patterns by epidemiologic phase, conducted supplementary within-sample hourly normalization analyses, and modeled longitudinal symptom trajectories using ARIMA (autoregressive integrated moving average) and supplementary ARIMAX (autoregressive integrated moving average with exogenous regressors) time-series models.

**Results:**

Anxiety-related posts closely followed influenza activity, surging during the outbreak and peak phases and remaining elevated even after influenza declined. Initial Spearman correlation analyses showed significant negative associations for irritability (*r*=−0.413; *P*=.02) and restlessness or feeling keyed up or on edge (*r*=–0.396; *P*=.02). However, supplementary ARIMAX analyses further revealed that being easily fatigued and difficulty concentrating or mind going blank exhibited more stable positive temporal associations with influenza activity after controlling for autocorrelation and lagged effects. Diurnal patterns shifted across stages, showing mild early-evening variation during the outbreak, clear morning peaks with secondary afternoon and evening rises during prevalence and decline, and morning-afternoon concentration in the end stage. Supplementary within-sample hourly normalization analyses showed that the major diurnal structures remained generally stable after normalization. ARIMA time-series analysis revealed that irritability and being easily fatigued consistently dominated the discussions, whereas others remained at relatively low levels. Out-of-sample forecasting based on a chronological 80% training and 20% testing split suggested generally stable short-term trajectories, with being easily fatigued showing a slight increase.

**Conclusions:**

This study demonstrates how social media can capture diurnal and seasonal fluctuations of anxiety symptoms associated with influenza activity, advancing understanding of affective dynamics in population health.

## Introduction

In the digital era, the collection, use, and analysis of social media data have created new opportunities across diverse domains. For example, it can be used to monitor public opinion, track consumer behavior, and follow emerging social issues. In the field of mental health, social media likewise provides a novel pathway for the early identification of problems. Given the limited accessibility of traditional mental health services and the reluctance of many individuals to actively seek professional help when experiencing psychological distress, exploring alternative approaches to detection and intervention is particularly important. The emergence of social media platforms offers a promising avenue to address this challenge [1].

Social media has become an important channel for individuals to express emotions [2,3], document daily life, and reflect psychological states [4], with its texts often embedding psychological cues such as emotional fluctuations, cognitive biases, and social behavior patterns [4-9]. For instance, De Choudhury et al [5], using Twitter data, demonstrated that users’ linguistic features and behavioral patterns can reveal their psychological states, highlighting the potential of social media texts for understanding depressive symptoms.

Subsequent studies have extended this line of research. Zhu et al [8] and Yu et al [7] analyzed discussions on anxiety and depression disorders on Sina Weibo and identified their linguistic features, further supporting the capacity of social media data to reflect collective perceptions and attitudes toward mental health issues.

Compared with traditional surveys or clinical interviews, social media data offer unique advantages for identifying linguistic markers of public mental health: (1) real-time monitoring, which captures daily fluctuations; (2) naturalistic expression, which reduces social desirability bias; and (3) accessibility, as platform APIs enable large-scale and longitudinal tracking [10]. These features make social media an important complement to conventional screening tools and provide new possibilities for the early identification of mental health problems [11].

Some early studies have demonstrated this potential. For example, Hung and Tadius [12] used Twitter data labeled as “depressed” and “non-depressed” to compare various machine learning methods for distinguishing between different types of texts. The results showed that one approach achieved superior accuracy and was able to automatically detect potential depression-related expressions in users’ writing. This suggests that social media text data can serve as linguistic markers of mental health symptoms [4].

Mental health symptoms, particularly anxiety, exhibit pronounced temporal dynamics. Anxiety does not emerge instantaneously but rather unfolds through processes of accumulation, fluctuation, and recovery in response to environmental changes [13]. Accordingly, longer observation periods are essential to reveal its developmental mechanisms and interactions with contextual factors. Among the possible temporal frameworks, the influenza season offers unique advantages. Unlike transient social events, influenza seasons typically last for several months and proceed through distinct stages, from preoutbreak to recession. This cyclical progression provides a natural context for examining the dynamic shifts in linguistic markers and exploring the interplay between infectious disease outbreaks and collective anxiety-related expressions. In the Chinese context, the 2023‐2024 influenza season holds significance that extends beyond traditional seasonal influenza research. First, it represents the first complete influenza cycle following China’s full withdrawal of nonpharmaceutical interventions against COVID-19. The strict social distancing and lockdown policies implemented during the pandemic [14,15] markedly reduced influenza incidence and population immunity between 2019 and 2022 [16], thereby heightening the intensity and health risks of this influenza season compared with previous years. Second, in the postpandemic phase of psychological recovery, the public may perceive influenza—a disease that is both familiar and threatening—in novel ways, potentially eliciting distinctive patterns of cognition and emotional response [17]. This psychological tension, situated between normalized disease risks and the residual memory of a global health crisis, makes the 2023‐2024 influenza season an ideal setting for investigating the dynamic evolution of anxiety symptoms [18]. In sum, this unique context offers not only a rare opportunity to examine how collective psychology adapts to new large-scale health threats in the aftermath of COVID-19 but also valuable empirical evidence to inform public health interventions.

Existing literature suggests a close association between influenza and anxiety. Wang and Sabran [19], in a survey conducted during the 2023‐2024 influenza season, found that the anxiety levels of young individuals were significantly associated with the impact of influenza on physical and mental health as well as quality of life. Similarly, Bults et al [20] reported that during the early phase of the 2009 H1N1 outbreak, public anxiety levels were relatively high but declined over time. Tausczik et al [21], through analyses of textual and browsing behaviors, further showed that the use of anxiety-related words surged at the onset of the pandemic and quickly subsided thereafter. However, most existing studies have focused primarily on the 2009 H1N1 influenza outbreak, while research on the 2023‐2024 influenza season remains scarce. In addition, prior studies often relied on cross-sectional data or concentrated on peak periods, lacking longitudinal tracking across the recurring stages of the influenza season. Consequently, 2 major limitations emerge: first, the inability to capture the temporal dynamics of emotional changes; and second, the restricted capacity for causal inference, making it difficult to determine whether fluctuations in anxiety levels are directly attributable to influenza itself or influenced by other environmental factors.

Building on this, this study adopts a longitudinal perspective to systematically investigate the evolution of public anxiety symptoms during the 2023‐2024 influenza season. We collected Sina Weibo data from September 4, 2023, to April 28, 2024, covering the entire influenza cycle—thereby enabling us to comprehensively trace the dynamic changes in anxiety symptoms across different stages through linguistic markers and to analyze their intrinsic connection with the course of influenza.

In addition, this study innovatively incorporates a diurnal variation perspective to provide a more comprehensive understanding of the temporal dynamics of anxiety symptoms. Existing research has shown that diurnal fluctuations are closely related to anxiety [22-26]. Individual chronotypes, reflecting differences in diurnal preferences, further shape these patterns [26,27]. For instance, Cox et al [25], using high-frequency tracking, found that anxiety levels exhibit pronounced within-day variation and differ between morning-type and evening-type individuals. Although based on limited samples, these findings indicate that anxiety symptoms fluctuate along diurnal cycles.

Similar patterns are also reflected in online contexts. For instance, Ten Thij et al [28] observed that individuals with depression expressed significantly more negative emotions—such as hopelessness, anxiety, and sadness—on social media during the midnight-to-early-morning hours, reflecting the psychological stress and emotional distress they experienced at night. However, most of these studies have focused on depression or comorbid mental health symptoms and disorders, and evidence specifically addressing diurnal variation in anxiety symptoms remains limited. Therefore, exploring the diurnal patterns of anxiety during the influenza season not only deepens our understanding of the temporal dynamics of anxiety but also helps fill gaps in the existing literature.

In summary, this study seeks to advance the field through 3 main contributions. Rather than identifying psychological effects of influenza or establishing predictive mental health signals, the study provides a descriptive temporal analysis of anxiety-related linguistic expressions on social media during a large-scale public health period and aims to characterize how anxiety-related expressions fluctuate across influenza stages and diurnal periods. First, it integrates large-scale social media data with the phased progression of the influenza season to longitudinally trace the dynamics of anxiety symptoms. Second, it introduces a novel diurnal perspective to examine the temporal expression of anxiety. Third, it applies time-series modeling to describe temporal covariation patterns between anxiety-related expressions and influenza activity. Accordingly, the specific research questions are as follows:

- RQ1: How are public anxiety symptoms dynamically associated with influenza incidence during the 2023-2024 influenza season?
- RQ2: During the 2023-2024 influenza season, how do public anxiety symptoms change in terms of diurnal rhythm patterns?
- RQ3: What are the evolutionary trends of different categories of anxiety symptoms over the course of the 2023-2024 influenza season?

By addressing the above questions, this study not only fills gaps in the existing literature but also provides empirical support for public health interventions and real-time mental health monitoring, underscoring the critical value of social media in mental health research [4].

## Methods

### Overview

This study used multiple steps: first, Weibo anxiety symptom data were cleaned and preprocessed. Second, statistical analysis and visualization indicated anxiety symptom diurnal trends. Third, anxiety symptom progression and influenza incidence rates were examined dynamically. Finally, time-series analysis revealed long-term anxiety symptom dynamics, providing novel research insights.

### Data Collection and Preprocessing

The description of generalized anxiety disorder in *Diagnostic and Statistical Manual of Mental Disorders*, Fourth Edition (*DSM-IV*) indicates that anxiety symptoms are primarily manifested in the following aspects: (1) restlessness or feeling keyed up or on edge, (2) being easily fatigued, (3) difficulty concentrating or mind going blank, (4) irritability, (5) muscle tension, and (6) sleep disturbance[29]. We selected these terms, translated them into Chinese, and used them as search keywords, as shown in Table 1.

Therefore, we selected specific terms, translated them into Chinese, and used them as search keywords. We crawled all Weibo posts containing these keywords from September 4, 2023, to April 28, 2024, fine-grained on an hourly basis, resulting in 169,728 posts as the raw data.

Data were collected using a Python-based web crawler that retrieved publicly accessible Sina Weibo posts through keyword-based searches. Keyword matching was based on substring matching rather than exact phrase matching. Only posts primarily written in simplified Chinese were retained. All timestamps were converted to China Standard Time (UTC+8) before hourly aggregation. Exact duplicate posts and reposted or highly repetitive irrelevant content were removed during preprocessing.

To examine the textual features and thematic structures of the posts, we applied Python’s re module to remove non-Chinese elements through iterative steps: deleting emojis, Unicode special characters, user mentions (@), hashtags (#), special markers (eg, 【】 and “fold”), redundant whitespace, duplicates, and overly short posts.

**Table 1.** Keywords to web crawl^a^.

Symptoms name	Translation in Chinese
Restlessness or feeling keyed up or on edge	焦躁不安, 感到紧张不安
Being easily fatigued	容易疲劳
Difficulty concentrating or mind going blank	注意力难以集中, 脑子一片空白
Irritability	易怒
Muscle tension	肌肉紧张
Sleep disturbance	睡眠障碍

a To improve linguistic coverage, approximately 200 candidate synonymous expressions across the 6 generalized anxiety disorder symptom categories were initially compiled. A supplementary pilot test using 10 synonymous expressions per category over a 3-day period (January 1‐3, 2024) yielded 57,062 posts after basic deduplication, illustrating the substantial increase in dataset complexity associated with exhaustive synonym expansion (Multimedia Appendix 1). This approach prioritized conceptual consistency with *Diagnostic and Statistical Manual of Mental Disorders* (*DSM*)–based symptom categories rather than exhaustive coverage of all colloquial expressions and was intended to capture population-level temporal patterns rather than identify clinical anxiety at the individual level.

All preprocessing rules were predefined before the main statistical analyses and applied consistently across the full dataset. The iterative cleaning process primarily involved refinement of rule clarity and quality checks to improve consistency between research assistants, rather than introducing new selection criteria based on observed temporal patterns or study results. Core inclusion and exclusion criteria remained stable throughout the process and were not adjusted for specific periods or outcomes.

Since automated preprocessing alone could not sufficiently filter out highly similar or irrelevant posts, an additional manual verification step was conducted. Additional details of the data collection and preprocessing procedures are provided in Multimedia Appendix 1. Manual verification primarily focused on whether posts reflected genuine anxiety-related expressions rather than metaphorical, commercial, or contextually unrelated uses of the keywords. Manual verification was primarily used as a quality-control step to assess consistency of rule implementation rather than to selectively modify the dataset based on study findings.

Frequency analysis was used to exclude high-frequency posts and irrelevant topics such as commercial advertisements, celebrity promotions, astrology, or unrelated events (eg, World Sleep Day). After several rounds of manual cleaning, we obtained a high-quality dataset of 106,440 posts.

Detailed preprocessing procedures, rule descriptions, and quality-control workflows are provided in Multimedia Appendix 2 to improve transparency and reproducibility.

### Ethical Considerations

This study used only publicly accessible Sina Weibo posts and did not involve interaction with human participants or access to private identifiable information. Before conducting the study, the research team consulted the university regarding ethics requirements. Based on the institutional guidance in effect at that time, the study was determined to be exempt from ethics review because it analyzed only publicly available, anonymized social media data. Therefore, institutional review board approval and informed consent were not required. To further protect participant privacy, all collected data were anonymized during preprocessing. No personally identifiable information, including user IDs or IP addresses, was collected or stored during the data collection process.

### Analysis Plan

#### RQ1: Step 1

We first collected and visualized weekly influenza data and anxiety symptom posting frequencies to organize and analyze dynamic changes in anxiety symptom data during the influenza season and identify the underlying correlations with influenza incidence.

PyCharm (JetBrains sro) and Python (version 3.12, Python Software Foundation; os, pandas, matplotlib, datetime, and seaborn) were used. After reading the Microsoft Excel file, we translated the “Post Publication Time” timestamp strings into “Week” and “Date” columns for analysis. We then analyzed and visualized the following:

- We counted weekly anxiety symptom posts by “Week” and “Search Criteria.”
- Using data from the *Influenza Weekly Report* of the Chinese National Influenza Center [30], we computed and graphed the influenza A positivity rate (Multimedia Appendix 3).
- We used matplotlib and seaborn to construct line charts to compare and depict the weekly posting frequencies of anxiety symptoms and influenza positivity rates over time.

Furthermore, to examine the relationship between influenza positivity rates and the posting frequency of various anxiety symptoms, we conducted a Spearman rank correlation analysis in R (version 4.4.1; R Foundation for Statistical Computing, with the *psych* package installed). This method was used to assess the monotonic relationships between variables and to reduce the influence of outliers, given the relatively small sample size (32 weeks). Statistical significance was determined at the threshold of *P*<.05.

To further examine the temporal associations between influenza positivity rates and anxiety-related symptom expressions, while controlling for autocorrelation and potential lag effects within the time series, we additionally constructed ARIMAX (autoregressive integrated moving average with exogenous regressors) models by incorporating influenza positivity rates as exogenous variables.

The ARIMAX analyses were conducted in R (version 4.4.1) using the auto.arima() function from the *forecast* package. Optimal model orders (p, d, q) were automatically selected based on the Akaike information criterion. Stationarity was assessed using the augmented Dickey-Fuller (ADF) test prior to modeling (Multimedia Appendix 4). As the analyses were based on weekly-level data, the time-series frequency was set to 52. No additional logarithmic or Box-Cox transformations were applied to the raw data [31].

To evaluate potential lagged effects, influenza positivity rates at contemporaneous (lag 0), 1-week lag (lag 1), and 2-week lag (lag 2) intervals were separately incorporated into the models. Model validation was performed using a chronological train or test split, with the first 80% of the data used for training and the remaining 20% reserved as an unseen test set [32]. The auto.arima() procedure for model order selection and parameter estimation was conducted exclusively on the training set, while the test set was used solely for out-of-sample evaluation. Regression coefficients of the exogenous variables and their corresponding 95% CIs were subsequently extracted to construct coefficient effect plots, illustrating the direction, strength, and statistical significance of the associations between influenza positivity rates and anxiety-related symptom expressions under different lag conditions.

#### RQ2: Step 2

The second step was to divide the influenza process into 3 stages and analyze the diurnal fluctuations of 6 anxiety symptoms within each stage.

Based on turning points in influenza A positivity rates reported by Chinese National Influenza Center weekly surveillance, we defined the outbreak stage as November 6‐26, 2023, when positivity began to rise sharply; the prevalence-decline stage as November 27, 2023, to February 29, 2024, when positivity remained high before declining; and the end stage as March 1 to April 28, 2024, when positivity returned to baseline [17]. Additionally, we investigated the number of anxiety-related posts per 0‐24 hours in different stages. These steps are specific:

- Data preprocessing: We read the Excel data file from the provided directory, transformed the “post publication time” column time strings to timestamps, and then sorted by hour to count posts in each group.
- Data statistics: We segmented the data by 1‐24 hours to count the number of posts for each anxiety symptom, and then summarized the data for the 3 phases to get the overall number of posts in each stage.
- Chart optimization and visualization: We set Matplotlib font options to display Chinese characters correctly, and then drew line charts of diurnal post quantity changes in Seaborn to intuitively portray anxiety symptom post variation trends.

To further examine whether the observed circadian patterns might be influenced by hourly fluctuations in posting activity within the anxiety-related dataset, we additionally conducted a robustness analysis using within-sample hourly normalization [32]. Specifically, within each influenza stage and corresponding hour, the number of posts for a given anxiety symptom category was expressed as the proportion of all anxiety-related posts identified during the same hour. For example, if 100 anxiety-related posts were identified within a given hour and 30 of them belonged to the “irritability” category, the relative expression frequency of irritability for that hour was calculated as 30%.

This within-sample normalization approach helps control, to some extent, for hourly fluctuations in posting activity within the anxiety-related dataset and was used to assess whether the observed circadian patterns remained stable after normalization. The normalized circadian variation plots are provided in Multimedia Appendix 5.

PyCharm was used to write and run all code, assuring analytical efficiency and reproducibility.

#### RQ3: Step 3

The steps are as follows:

1. Data preprocessing: Timestamps were converted to date format using the as.date () function from the *tidyverse* package, along with the *readxl* package.
2. Generating time-series data: The number of posts for each category was counted by date, and time-series data were generated using the group_by() and summarise() functions.

To characterize the temporal evolution of different anxiety symptoms during the influenza season, we used ARIMA (autoregressive integrated moving average) models for time-series analysis and short-term forecasting. The analyses were conducted in R (version 4.4.1) using the *forecast*, *xts*, and *ggprism* packages. First, timestamp data were converted into date format using the *tidyverse* and *readxl* packages, and posting counts for different anxiety symptom categories were aggregated by date to generate time-series data.

ARIMA models were fitted using the auto.arima() function from the *forecast* package, with optimal model orders (p, d, q) automatically selected based on the Akaike information criterion. The model effectively captures autocorrelated structures in time-series data, enables residuals to approximate white noise, and provides statistically interpretable short-term forecasts [32]. Prior to modeling, stationarity was assessed using the ADF test (Multimedia Appendix 6). The ARIMA models were constructed using daily-level data, with the time-series frequency set to 7. No additional transformations were applied to the raw data [32].

The time-series data were chronologically divided into training and testing sets, with the first 80% used for model training and the remaining 20% reserved as an unseen test set. Model order selection and parameter estimation using the auto.arima() function were performed exclusively on the training set, while the test set was used solely for out-of-sample evaluation of forecasting performance. In addition, a fixed-window backtesting strategy was applied to assess model robustness. Forecasting performance was evaluated using root mean square error, mean absolute error, mean absolute percentage error, and mean absolute scaled error. Furthermore, the ARIMA models were used for 30-day short-term forecasting to describe the potential temporal trajectories of different anxiety symptom categories [32].

## Results

### RQ1: The Relationship Between Posting Volumes of Various Anxiety Symptoms and Influenza Infection Rates

#### Overview

**Figure 1.**
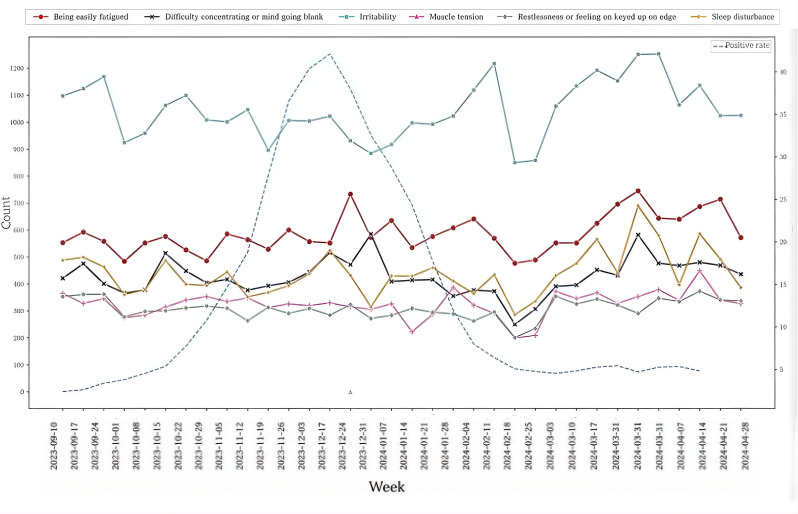
The relationship between influenza positive rate and 6 kinds of symptoms posts.

Figure 1 shows that during the 2023‐2024 influenza season, different anxiety symptoms exhibited distinct characteristics but overall maintained relatively moderate fluctuation patterns. Most symptoms remained at moderate levels from September 2023 to March 2024, with a downward trend observed in April 2024. Irritability and being easily fatigued were consistently the 2 most frequently mentioned symptoms, showing a fluctuating upward trend starting in late November and peaking at the end of January. Even after influenza positivity declined, these symptoms remained at relatively high levels. Sleep disturbance fluctuated repeatedly between November and March without forming a single peak, while difficulty concentrating or mind going blank showed a slight increase around December and another minor fluctuation in March. In contrast, muscle tension and restlessness or feeling keyed up or on edge remained at the lowest overall levels with limited variation.

#### Temporal Trend and Correlation Analysis

To further examine the above trends, we conducted a Spearman rank correlation analysis (Table 2). The results showed that only irritability (Spearman *r*=–0.413; *P*=.02) and restlessness or feeling keyed up or on edge (Spearman *r*=–0.396; *P*=.02) exhibited significant negative correlations with influenza positivity rates. Although the overall Spearman correlation between influenza positivity and irritability was negative (*r*=–.413; *P*=.02), irritability increased in parallel with influenza positivity during the outbreak and peak periods. However, irritability remained elevated even after influenza positivity declined, suggesting a lagged and persistent psychological response. This temporal mismatch may partially explain why the overall Spearman correlation coefficient appeared negative across the full study period. The other symptoms, while showing some local consistency with influenza fluctuations, did not reach statistical significance in their correlation coefficients.

**Table 2.** Results of Spearman rank correlation analysis between influenza positivity and anxiety symptom postings.

Symptom name	*r*	*P* value	95% CI
Irritability	–0.413	.02	–0.659 to –0.086
Difficulty concentrating or mind going blank	0.128	.47	–0.220 to 0.447
Restlessness or feeling keyed up or on edge	–0.396	.02	–0.647 to –0.066
Sleep disturbance	–0.221	.21	–0.520 to 0.127
Being easily fatigued	0.093	.60	–0.252 to 0.418
Muscle tension	–0.225	.20	–0.523 to 0.123

#### Robustness Analysis: ARIMAX Analysis of Temporal Associations

To further control for autocorrelation and potential lag effects within the time series, we additionally constructed ARIMAX models using influenza positivity rates as exogenous variables, incorporating contemporaneous (lag 0), 1-week lag (lag 1), and 2-week lag (lag 2) conditions separately. Compared with Spearman correlation analysis, ARIMAX models are better able to capture the dynamic temporal associations between influenza activity and anxiety-related symptom expressions. Detailed ARIMAX coefficient estimates are presented in Table 3, while full ARIMAX model specifications, including model orders (p, d, q), ADF stationarity test results, and diagnostic statistics, are provided in Multimedia Appendix 4.

**Table 3.** ARIMAX^a^ results for anxiety-related symptom expressions under different lag conditions.

Symptom name and lag_model	Lag	β	*P* value	95% CI
Irritability				
lag0_concurrent	0	–3.0348	.08	–6.4734 to 0.4038
lag1_one_week	1	-3.1407	.10	–6.5525 to 0.2711
lag2_two_weeks	2	-3.0325	.08	–6.4384 to 0.3734
Difficulty concentrating or mind going blank				
lag0_concurrent	0	2.0798	.05	0.0308 to 4.1288
lag1_one_week	1	2.1684	.01	0.5218 to 3.815
lag2_two_weeks	2	1.9615	.06	–0.101 to 4.024
Restlessness or feeling keyed up or on edge				
lag0_concurrent	0	–0.412	.56	–1.7999 to 0.9759
lag1_one_week	1	–0.5406	.43	–1.8973 to 0.8161
lag2_two_weeks	2	–0.6861	.31	–2.0048 to 0.6326
Sleep disturbance				
lag0_concurrent	0	–0.2284	.88	–3.2556 to 2.7988
lag1_one_week	1	–0.3805	.80	–3.3695 to 2.6085
lag2_two_weeks	2	–0.469	.76	–3.4443 to 2.5063
Being easily fatigued				
lag0_concurrent	0	1.4068	.06	–0.0473 to 2.8609
lag1_one_week	1	1.7447	.01	0.3574 to 3.132
lag2_two_weeks	2	1.8699	.007	0.5157 to 3.2241
Muscle tension				
lag0_concurrent	0	–0.1324	.88	–1.8923 to 1.6275
lag1_one_week	1	–0.3161	.72	–2.0574 to 1.4252
lag2_two_weeks	2	–0.4388	.62	–2.1669 to 1.2893

a ARIMAX: autoregressive integrated moving average with exogenous regressors.

**Figure 2.**
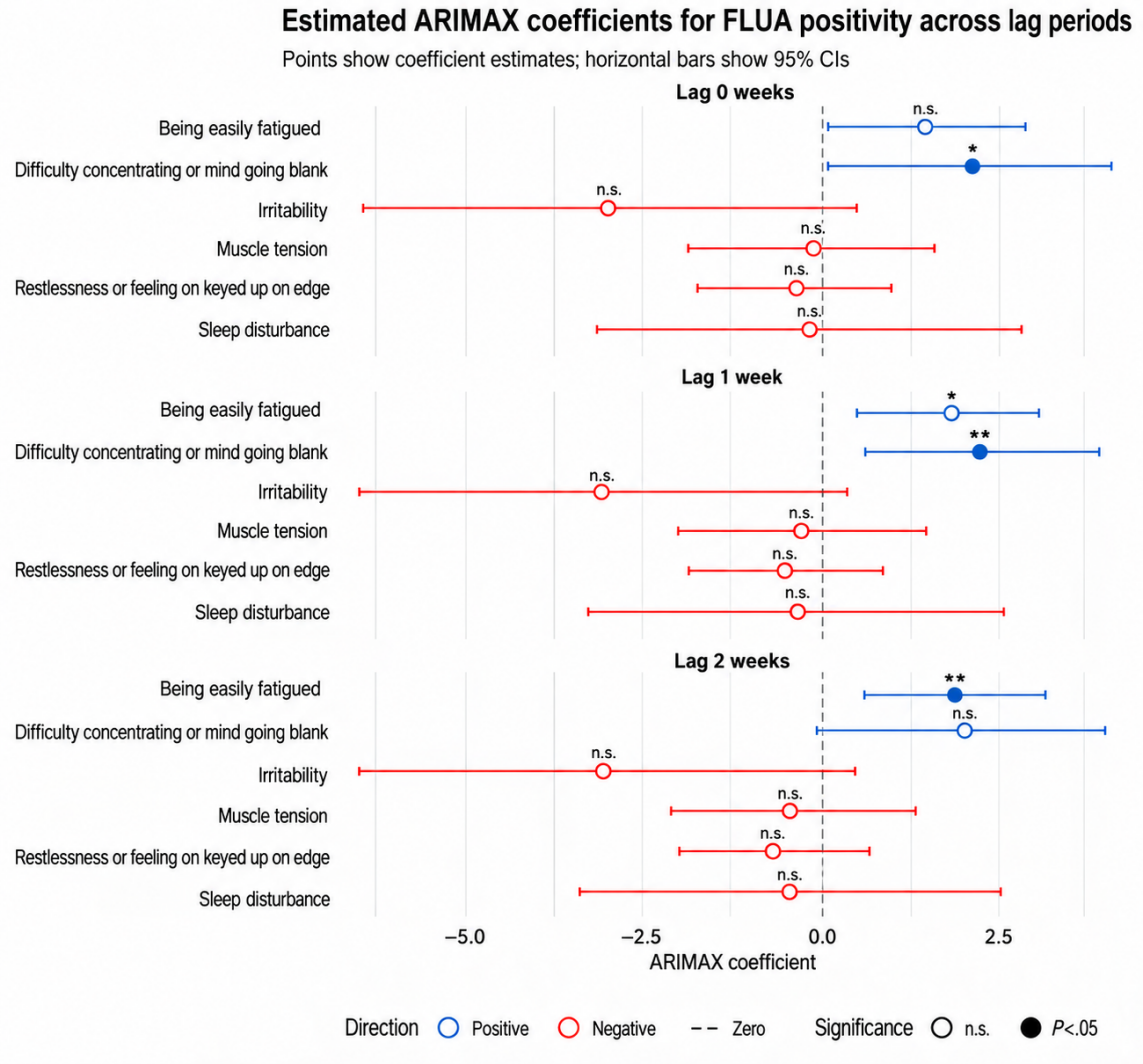
ARIMAX coefficient estimates under different lag conditions. ARIMAX: autoregressive integrated moving average with exogenous regressors; FLUA: Influenza A virus; n.s.: not significant.

The results showed that, after controlling for temporal dependency structures, the association patterns between different anxiety symptoms and influenza positivity rates differed substantially (Figure 2). In the contemporaneous model (lag 0), influenza positivity rates were significantly positively associated with difficulty concentrating or mind going blank, whereas being easily fatigued showed a positive trend that did not reach statistical significance. The remaining symptoms, including irritability, sleep disturbance, muscle tension, and restlessness or feeling keyed up or on edge, generally did not exhibit significant associations.

In the 1-week lag model (lag 1), influenza positivity rates were significantly positively associated with both being easily fatigued and difficulty concentrating or mind going blank, with the strength of association for difficulty concentrating or mind going blank becoming further enhanced. These findings suggest that certain anxiety-related expressions may not occur entirely synchronously with influenza activity, but instead may reflect delayed psychological or behavioral responses following peaks in influenza activity.

In the 2-week lag model (lag 2), being easily fatigued remained significantly positively associated with influenza positivity rates, whereas difficulty concentrating or mind going blank, although still showing a positive trend, no longer remained statistically significant. Meanwhile, the remaining symptoms did not demonstrate stable significant temporal associations across different lag conditions.

Overall, the ARIMAX findings differed somewhat from the Spearman correlation results. Spearman analyses indicated significant negative correlations between influenza positivity rates and both irritability and restlessness or feeling keyed up or on edge. However, after controlling for autocorrelation and lag effects, the ARIMAX models did not identify stable significant associations for these symptoms. In contrast, the ARIMAX analyses further revealed more stable positive temporal dynamic associations between influenza activity and both being easily fatigued and difficulty concentrating or mind going blank, particularly within the lagged models.

These findings suggest that simple correlation analyses may be influenced by overall temporal trends and stage-specific fluctuations, whereas ARIMAX models, after accounting for time-series dependency structures, are better able to capture the dynamic relationships between influenza activity and anxiety-related expressions. Although the 2 analytical approaches differed regarding which specific symptoms reached statistical significance, both overall supported the core conclusion of this study: anxiety-related expressions dynamically fluctuated alongside influenza activity during the influenza season and exhibited certain persistent and stage-dependent characteristics.

### RQ2: Diurnal Variation Chart of Posting Volumes for Various Anxiety Symptoms

#### Overview

**Figure 3.**
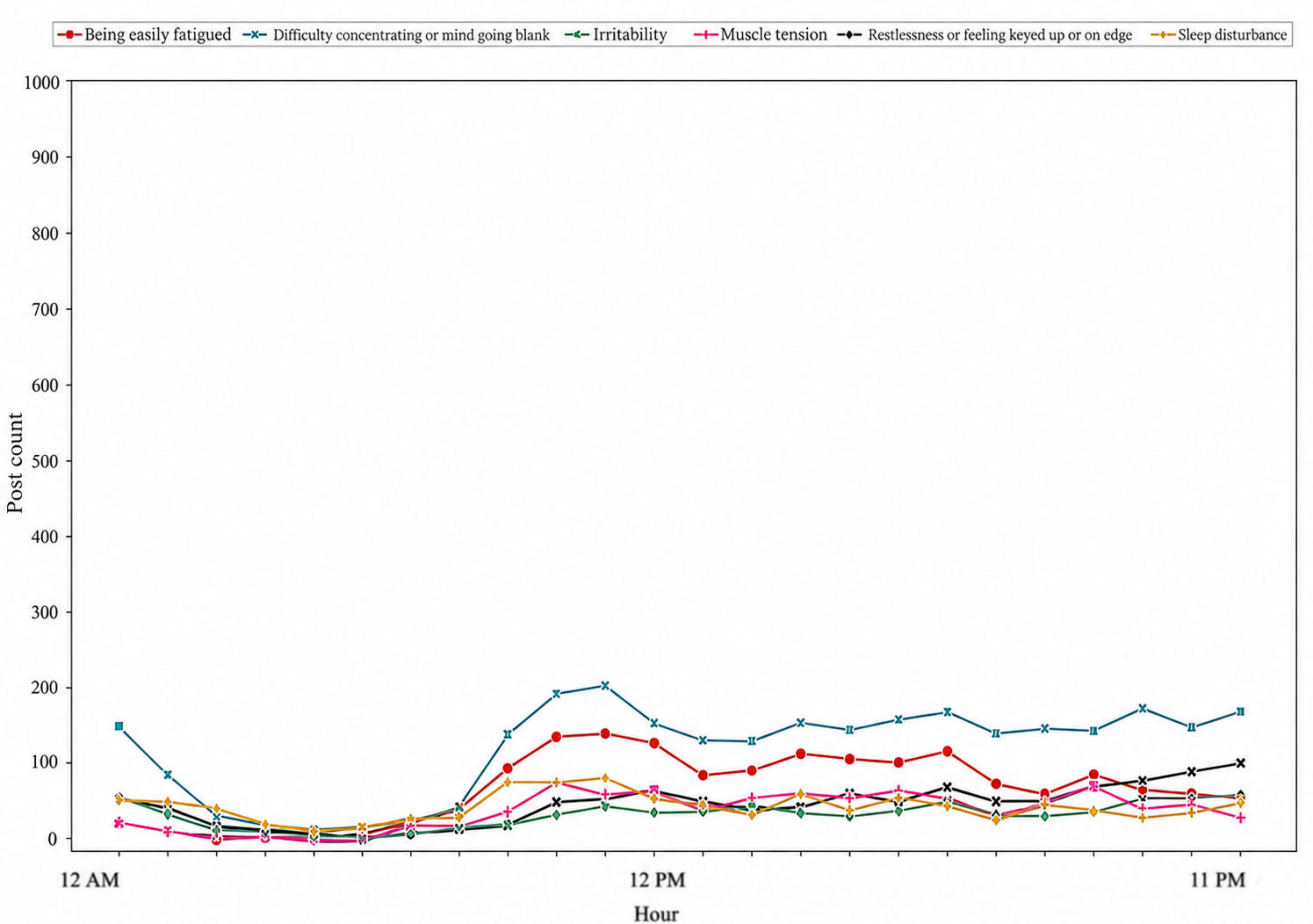
Diurnal variation chart of posting volumes for various anxiety symptoms of the first stage.

**Figure 4.**
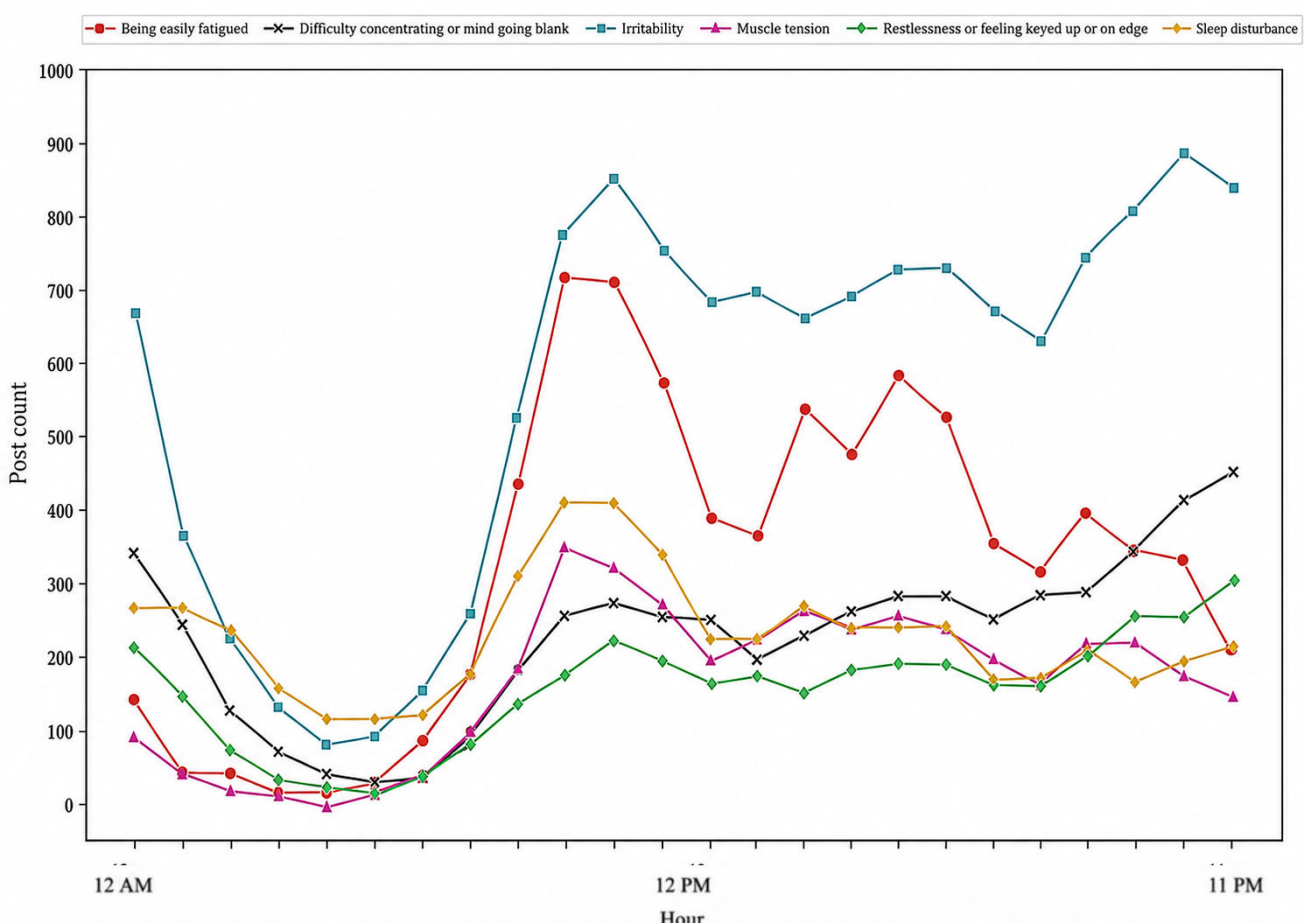
Diurnal variation chart of posting volumes for various anxiety symptoms of the second stage.

**Figure 5.**
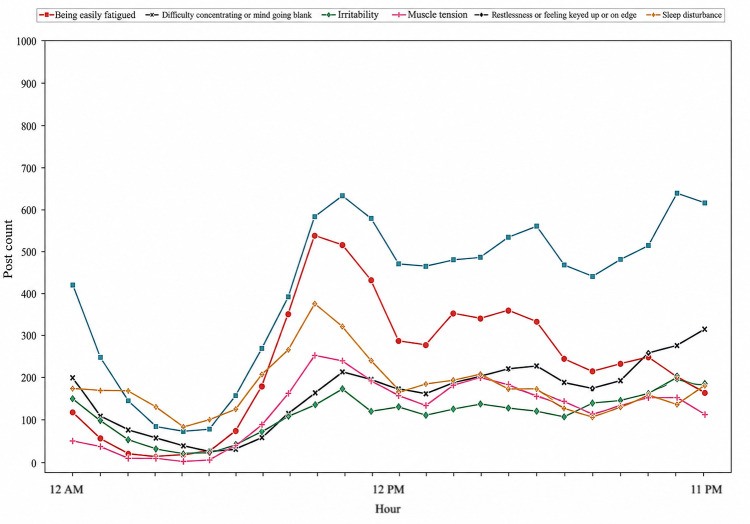
Diurnal variation chart of posting volumes for various anxiety symptoms of the third stage.

Figures 3-5 show the total number of linked posts for each phase in each 0‐ to 24-hour period determined using Excel and Python.

#### Phase I: The Influenza Outbreak Stage (November 6, 2023, to November 26, 2023)

In terms of diurnal distribution, irritability and being easily fatigued peaked in the morning (9-10 AM), while difficulty concentrating or mind going blank showed a slight increase during nighttime, and the overall fluctuations in symptom expression were relatively modest throughout the outbreak stage.

#### Phase II: The Influenza Prevalent and Declining Stage (November 27, 2023, to February 29, 2024)

During this stage, the diurnal distribution exhibited a clearer pattern. Symptom expression was most concentrated in the morning (9-11 AM), with relative secondary peaks in the afternoon (4-5 PM) and evening (9-11 PM). Among them, irritability and being easily fatigued remained relatively prominent across different time periods, whereas changes in other symptoms were comparatively limited.

#### Phase III: The End of Influenza Season (March 1, 2024, to April 28, 2024)

After the influenza season ended, the diurnal distribution became more simplified, with symptom expression mainly concentrated in the morning (9-11 AM) and afternoon (4-5 PM), and fewer symptoms expressed in the evening. Irritability and being easily fatigued remained at relatively higher levels within the daily distribution, but the gap with other symptoms diminished.

The diurnal curves in this study illustrated relative distribution patterns within each stage, rather than absolute posting levels across stages; therefore, overall platform activity was not used as a baseline. Across stages, irritability and being easily fatigued consistently emerged as the most prominent symptoms. Morning was generally the period with the highest concentration of symptom expression, while secondary peaks appeared in the afternoon or evening in certain stages, a common feature throughout the influenza season.

#### Robustness Analysis: Examination of Circadian Patterns Based on Within-Sample Hourly Normalization

To further examine whether the observed circadian patterns were primarily influenced by hourly fluctuations in posting activity within the anxiety-related dataset, we conducted an additional robustness analysis using within-sample hourly normalization. Specifically, within each influenza stage and corresponding hour, the number of posts for a given anxiety symptom category was divided by the total number of anxiety-related posts identified during the same hour, thereby generating the relative expression proportion of each symptom within that hour rather than using raw posting counts. This approach helps control, to some extent, for differences in posting volume across hours within the dataset and was used to assess whether the observed circadian patterns remained stable after normalization. The normalized circadian variation plots are provided in Multimedia Appendix 5.

The normalized results were generally consistent with the original count-based analyses, suggesting that the observed circadian patterns were not solely driven by hourly fluctuations in posting activity within the anxiety-related dataset. Across all 3 influenza stages, irritability and being easily fatigued remained the predominant symptom categories, with higher concentrations of expression still observed during daytime hours. Overall, the major temporal structures identified in the original analyses remained generally stable after normalization.

At the same time, the normalization analyses also revealed several localized differences. Compared with the raw-count results, sleep disturbance exhibited a more pronounced relative proportion during late-night and early-morning hours, particularly during the influenza outbreak and decline stages, suggesting that sleep-related expressions may become relatively more concentrated when overall posting activity is lower. In addition, some pronounced morning peaks observed in the original analyses became attenuated after normalization and instead appeared as broader daytime plateau patterns. This suggests that certain morning peaks may have been partially influenced by routine platform activity cycles, although the overall circadian temporal structure remained largely unchanged.

During the epidemic and decline stages, the normalized results continued to show relatively high proportions of irritability during daytime and evening hours, while being easily fatigued remained consistently elevated throughout daytime periods. Meanwhile, difficulty concentrating or mind going blank demonstrated a relatively increased proportion during evening hours, whereas the remaining symptoms generally maintained relatively stable temporal distribution patterns.

Overall, the robustness analyses largely supported the stability of the original findings, indicating that the observed circadian variation patterns were not simply driven by differences in posting activity within the dataset. At the same time, the normalization analyses further revealed more subtle differences in the relative expression of specific symptoms across particular time periods.

However, because hourly posting-volume data for the overall Sina Weibo platform were unavailable, strict platform-level normalization could not be performed. Therefore, the potential confounding influence of broader platform activity cycles cannot be completely excluded. Accordingly, the findings of this study should be interpreted as descriptive temporal patterns of anxiety-related expressions rather than prevalence estimates at the platform-wide population level.

### RQ3: The Evolution Trends of Various Anxiety Symptoms

The core objective of the ARIMA model is to extract the underlying correlation structure of the time series so that the residuals approximate white noise. Stationarity was assessed using the ADF test prior to model fitting. Residual autocorrelation analysis showed that the mean residuals of all symptom models were close to 0. Residual plots, autocorrelation function or partial autocorrelation function plots, and quantile-quantile plots (Multimedia Appendix 6) further confirmed that the residuals showed no systematic trends or outliers and were approximately normally distributed, thereby supporting the adequacy of the model fit. Meanwhile, Table 4 presents the predictive performance of the ARIMA time-series model, which was used to evaluate out-of-sample forecasting performance and model generalizability. Based on the chronological 80% training and 20% hold-out testing split, the out-of-sample prediction errors were within an acceptable range, suggesting that the ARIMA models demonstrated generally adequate short-term forecasting performance across symptom categories.

**Table 4.** Out-of-sample forecasting performance of ARIMA^a^ models across anxiety symptom categories^b^.

Symptom name	Model order(p,d,q)^c^	RMSE^d^	MAE^e^	MAPE^f^	MASE^g^	Ljung_Box, chi-square (*df*=10)	*P* value	White_noise
Irritability	ARIMA(2,0,0)(1,0,0) [7]	26.57	20.39	11.81	1.02	9.7	.47	Yes
Difficulty concentrating or mind going blank	ARIMA(0,1,2)(0,0,1) [7]	17.07	11.85	15.28	1.12	6.2	. .80	Yes
Restlessness or feeling keyed up or on edge	ARIMA(1,1,1)(0,0,1) [7]	7.89	6.6	14.59	0.8	8,7	.56	Yes
Sleep disturbance	ARIMA(1,0,0)(0,0,2) [7]	28.38	17.54	21.79	1.38	4,4	.93	Yes
Being easily fatigued	ARIMA(2,0,2)(1,0,0) [7]	28.02	20.21	19.56	1.32	6,0	.81	Yes
Muscle tension	ARIMA(2,0,0)(2,0,0) [7]	14.5	10.14	17.86	0.95	7.1	.72	Yes

a ARIMA: autoregressive integrated moving average.

b RMSE, MAE, MAPE, and MASE were calculated exclusively on the unseen test set to evaluate out-of-sample forecasting performance and model generalizability.

c Models with *d*=0 included an intercept (nonzero mean).

d RMSE: root mean square error.

e MAE: mean absolute error.

f MAPE: mean absolute percentage error.

g MASE: mean absolute scaled error.

**Figure 6.**
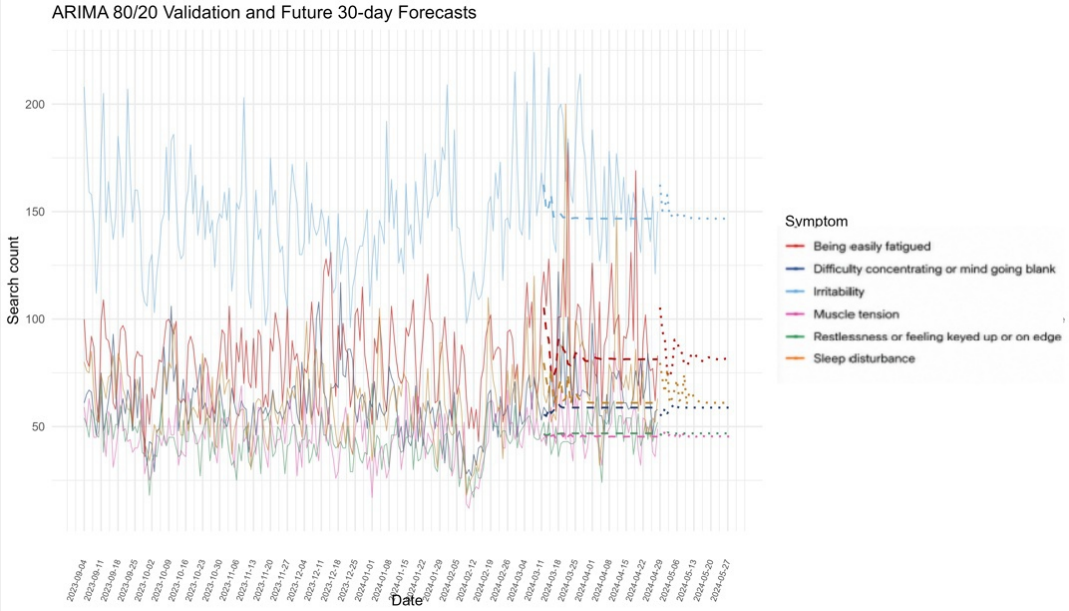
ARIMA forecasting of anxiety-related symptom expressions using an 80/20 train-test split. ARIMA: autoregressive integrated moving average.

As shown in Figure 6, discussions related to irritability and being easily fatigued were the most prevalent in the historical data, while other symptoms (such as sleep disturbance, difficulty concentrating or mind going blank, restlessness or feeling keyed up or on edge, and muscle tension) appeared at relatively low levels. Out-of-sample forecasting results based on the hold-out test set generally captured the major temporal patterns and fluctuations observed in the anxiety-related symptom series, thereby supporting the robustness of the short-term forecasts. The 30-day forecasting analysis further suggested that irritability would remain at its current level; being easily fatigued was generally stable but showed a slight increase; sleep disturbance and difficulty concentrating or mind going blank remained overall stable with only minor upward fluctuations; restlessness or feeling keyed up or on edge might show a slight decrease; and muscle tension was expected to remain relatively stable.

## Discussion

### Principal Findings

This study identified dynamic temporal patterns of anxiety-related expressions during the 2023‐2024 influenza season. Across analyses, anxiety-related expressions fluctuated alongside influenza activity and exhibited stage-dependent diurnal variation patterns. Supplementary ARIMAX and normalization analyses further suggested that several temporal associations and circadian structures remained relatively stable after accounting for lagged effects and within-sample posting fluctuations. In addition, ARIMA-based forecasting indicated generally stable short-term trajectories across symptom categories, with being easily fatigued remaining one of the most prominent symptoms over time.

Although keyword-based approaches may involve individual-level misclassification, the converging temporal patterns observed across correlation, time-series, and normalization analyses suggest that the extracted linguistic markers may capture meaningful population-level fluctuations in anxiety-related expressions rather than purely random noise. Nevertheless, these findings should be interpreted as descriptive temporal patterns of anxiety-related expressions within a public health context rather than evidence of causal psychological effects.

### Theoretical Significance

#### Linguistic Markers on Social Media Linking Anxiety Symptoms With Influenza Incidence

This study explores the dynamic characteristics of social media in tracking public mental health. The results (step 1) suggest that anxiety-related expressions on social media exhibited dynamic temporal associations with influenza activity during the 2023‐2024 influenza season. Initial Spearman correlation analyses showed significant negative correlations for irritability and restlessness or feeling keyed up or on edge, whereas other symptoms demonstrated nonsignificant trends. However, supplementary ARIMAX analyses further revealed that, after controlling for temporal autocorrelation and lagged effects, these negative associations were no longer stable. Instead, being easily fatigued and difficulty concentrating or mind going blank exhibited more consistent positive temporal associations with influenza activity, particularly under lagged conditions. This discrepancy suggests that simple correlation analyses may not fully capture the temporal complexity of anxiety-related expressions during influenza seasons, whereas time-series approaches are better able to identify delayed and dynamic anxiety-related expressions associated with influenza activity.

In addition, anxiety-related posts remained elevated until April 2024, when they began to decline as temperatures rose and the influenza season came to an end, suggesting that anxiety-related expressions during influenza seasons may not fluctuate in complete synchrony with influenza positivity rates and may also be influenced by daily routines, seasonal variations, and broader social adjustment processes. These results demonstrate that social media tracking not only reflects the immediate anxiety-related expressions to influenza but also captures the potential persistence of anxiety symptoms during the recovery phase.

Further examination of the temporal dynamics reveals local synchronization: during certain weeks, particularly around the peak influenza period, anxiety-related postings fluctuated in parallel with influenza activity. Between late November 2023 and the end of January 2024, the physical and psychological impacts of influenza were accompanied by an overall increase in anxiety-related postings, mainly reflected in irritability and being easily fatigued. The lagged associations identified in the ARIMAX models further suggest that certain anxiety-related expressions may emerge or persist after increases in influenza activity, indicating delayed and sustained psychological or behavioral responses rather than purely concurrent reactions.

Overall, the findings suggest that mental health tracking on social media is not static but dynamically adjusts to changes in public health conditions. During influenza outbreaks, public attention to health issues tended to increase, as did the online expression of anxiety symptoms [3]. Through social media, individuals promptly shared health experiences, expressed anxiety, and interacted with others, forming a collective coping mechanism [3]. Such dynamic participation supports individual mental well-being and contributes to a broader emotional support network at the societal level.

#### Linguistic Markers of Anxiety Symptoms’ Diurnal Patterns in Social Media

The research results (step 2) show that the diurnal expression patterns of anxiety symptoms exhibited dynamic changes across 3 phases of the influenza season—from the insignificant diurnal differences observed in the initial outbreak phase, to a concentration in the morning with smaller secondary peaks in the afternoon and evening during the prevalent and declining phase, and finally to a primary morning peak with a smaller secondary rise in the afternoon during the ending phase. This dynamic change indicates that social media tracking can sensitively capture subtle temporal differences in the expression of public anxiety symptoms. Supplementary within-sample hourly normalization analyses further demonstrated that the major diurnal structures remained generally stable after normalization, suggesting that the observed temporal patterns were unlikely to be explained entirely by hourly fluctuations in posting activity within the anxiety-related dataset.

As a real-time platform for the public to express emotions and health status, the linguistic marker nature of social media data enables us to precisely capture the different peak times of anxiety symptoms throughout the day [23,24]. For example, the morning peak of anxiety may be related to the stress of starting a new day, the afternoon secondary peak may reflect accumulated workload and social interaction–related tension, and the evening secondary peak may be associated with psychological fatigue and reduced self-regulation capacity after daily activities [33]. Although several peak intensities became attenuated after normalization, the overall morning and daytime concentration patterns persisted across influenza stages, further supporting the temporal stability of the observed diurnal structures. This dynamic change in diurnal rhythm shows that social media is a window reflecting individual biological rhythms and social activity patterns [34]. On the one hand, individual physiological rhythms (such as the sleep-wake cycle) may affect the frequency and timing of expressing anxiety on social media; on the other hand, the patterns of social activities (such as the timing of work, study, and social interactions) also influence individuals’ attention to and enthusiasm for discussing health issues. This dynamic change in diurnal rhythm further enriches the connotation of social media in mental health tracking, revealing behavioral characteristics and psychological needs at different times of the day [35].

#### Differential Evolutionary Trends of Anxiety Symptoms as Linguistic Markers on Social Media

This study found that, in both historical data and out-of-sample forecasting analyses based on a chronological 80% training and 20% testing split, irritability and being easily fatigued consistently dominated the discussions, underscoring the central role of emotional swings and physical fatigue in public anxiety experiences. The fitted results closely matched the original data, indicating that the time-series approach reliably captured historical trends. Based on the 30-day forecast, the overall trajectories are expected to remain stable, with being easily fatigued showing a slight increase. Although this change is modest, its persistent prominence across the historical period suggests that physical fatigue exerts a considerable impact on public experiences of anxiety during influenza seasons.

Therefore, as a widely prevalent disease with substantial physiological consequences, influenza not only imposes direct physical burdens but is also closely linked to public mental health [36-38]. These findings indicate that a dynamic tracking approach combining out-of-sample validation and short-term forecasting can effectively characterize the temporal evolution of anxiety symptoms during influenza seasons and offer methodological support for understanding their psychological and behavioral mechanisms as well as optimizing public mental health responses.

### Practical Implications

This study provides several practical implications for public health and mental health practice. First, the observed associations between influenza incidence and anxiety [37]-related discussions suggest that social media monitoring can serve as a descriptive observational resource of population mental health during public health events. During flu season, public mental health education and assistance reduce anxiety [3].

Second, the identified diurnal variations highlight the importance of time-sensitive strategies. Mental health campaigns and support services delivered during peak anxiety periods—particularly in the morning, with additional rises in the afternoon and evening—may enhance engagement and effectiveness [39].

Finally, the findings emphasize the need for sustained mental health monitoring during and after the influenza season to address delayed or lingering anxiety responses and promote long-term resilience [3,40].

### Limitations and Future Directions

This study has several limitations.

First, the use of Sina Weibo data may introduce sampling bias [4,41], limiting the generalizability of the findings to non-Weibo users [7].

Second, the keyword-based approach may introduce misclassification bias, as selected terms (eg, fatigue or sleep disturbance) may appear in nonanxiety contexts [32]. In addition, colloquial, metaphorical, or culturally specific expressions of anxiety (eg, slang terms) may not be fully captured. Future studies should incorporate context-sensitive AI methods [42], such as supervised classifiers or semantic embedding models, to improve measurement validity [43,44].

In addition, the keyword-based approach did not explicitly account for contextual linguistic phenomena such as negation, sarcasm, or irony. Therefore, posts containing anxiety-related keywords in nonanxious contexts may still have been included, which may affect classification accuracy. Accordingly, the findings should be interpreted as descriptive temporal patterns rather than direct measures of individual psychological states or clinical anxiety prevalence. Future studies should incorporate advanced context-sensitive natural language processing approaches, such as transformer-based models (eg, Bidirectional Encoder Representations from Transformers), to improve semantic understanding and classification accuracy.

Third, certain expressions such as “fatigue” may reflect multiple overlapping conditions or experiences, including work-related exhaustion, physical illness, sleep deprivation, or emotional distress, rather than anxiety specifically. This semantic ambiguity highlights the difficulty of inferring discrete psychological states from keyword-based social media data. Future studies should further incorporate contextual semantic analysis to better distinguish different sources and meanings of similar expressions.

Furthermore, given the large data volume, manual verification of each post was not feasible, and some irrelevant content may remain. Although some degree of misclassification may still exist, there is no clear reason to assume that these errors would systematically align with the temporal structures examined in this study (eg, influenza stages or diurnal cycles). Therefore, while potential bias cannot be completely excluded, the major temporal patterns observed are unlikely to be solely driven by preprocessing decisions.

In addition, this study did not control for potentially important contextual factors, such as holidays, academic schedules, media coverage, weather changes, or broader social events, which may also shape temporal fluctuations in anxiety-related expressions. Therefore, the findings should be interpreted as descriptive associations rather than evidence of causal inference.

Meanwhile, future research should address these issues by integrating multiplatform or survey data to reduce sampling bias, applying advanced natural language processing to capture informal expressions, and expanding the lexical framework beyond *DSM-IV* terms to reflect cultural nuances.

Finally, although supplementary within-sample hourly normalization analyses were conducted, the study was unable to perform full platform-level normalization because hourly posting volume data for the entire Weibo platform were unavailable. Therefore, residual confounding associated with broader platform activity patterns cannot be completely excluded. Future studies incorporating platform-wide posting volume as a baseline may help better distinguish true diurnal variations in anxiety-related expressions from general social media use rhythms.

### Conclusions

This study used social media data during the influenza season to examine diurnal patterns, dynamic fluctuations, and associations with influenza activity in public anxiety symptoms. The findings revealed influenza-related shifts in symptom expressions and differences across symptom categories, as reflected on social media.

Overall, the ubiquity of social media use provides valuable linguistic markers of affective symptoms [45], highlighting its potential value as a descriptive observational resource for understanding population mental health dynamics during infectious disease seasons [4].

## Supplementary material

10.2196/88849 Multimedia Appendix 1 Supplementary pilot analysis of synonymous anxiety-related expressions on Sina Weibo.

10.2196/88849 Multimedia Appendix 2 Detailed data preprocessing steps.

10.2196/88849 Multimedia Appendix 3 Positive rate of influenza A and percentage of influenza A among all positive cases.

10.2196/88849 Multimedia Appendix 4 Robustness analysis: autoregressive integrated moving average with exogenous regressors analysis of temporal associations.

10.2196/88849 Multimedia Appendix 5 Robustness analysis: examination of circadian patterns based on within-sample hourly normalization.

10.2196/88849 Multimedia Appendix 6 Details: residual plots, autocorrelation function or partial autocorrelation function plots, and quantile-quantile plots, and stationarity test of autoregressive integrated moving average.

## Abbreviations

ADF: augmented Dickey-Fuller
ARIMA: autoregressive integrated moving average
ARIMAX: autoregressive integrated moving average with exogenous regressors
*DSM-IV*: *Diagnostic and Statistical Manual of Mental Disorders*, Fourth Edition

## References

[1] Guntuku SC, Yaden DB, Kern ML, Ungar LH, Eichstaedt JC (2017). Detecting depression and mental illness on social media: an integrative review. Curr Opin Behav Sci.

[2] Oluwalade TI, Ahmadi H, Huo L, Sharpe R, Zhou SM (2025). Comorbidities and emotions—unpacking the sentiments of pediatric patients with multiple long-term conditions through social media feedback: a large language model-driven study. J Affect Disord.

[3] Zheng P, Adams PC, Wang J (2024). Shifting moods on Sina Weibo: the first 12 weeks of COVID-19 in Wuhan. New Media Soc.

[4] Wang R, Ji Z, Chen Y, Wu H, Liang X (2025). Identifying possible influence factors for depression through social media. J Affect Disord.

[5] De Choudhury M, Gamon M, Counts S, Horvitz E (2021). Predicting depression via social media. ICWSM.

[6] Jung H, Park HA, Song TM (2017). Ontology-based approach to social data sentiment analysis: detection of adolescent depression signals. J Med Internet Res.

[7] Yu L, Jiang W, Ren Z, Xu S, Zhang L, Hu X (2021). Detecting changes in attitudes toward depression on Chinese social media: a text analysis. J Affect Disord.

[8] Zhu J, Li Z, Zhang X, Zhang Z, Hu B (2023). Public attitudes toward anxiety disorder on Sina Weibo: content analysis. J Med Internet Res.

[9] Hofer M, Hargittai E (2024). Online social engagement, depression, and anxiety among older adults. New Media Soc.

[10] Skaik R, Inkpen D (2021). Using social media for mental health surveillance. ACM Comput Surv.

[11] Boursier V, Gioia F, Musetti A, Schimmenti A (2020). Facing loneliness and anxiety during the COVID-19 isolation: the role of excessive social media use in a sample of Italian adults. Front Psychiatry.

[12] Hung LP, Tadius EM Journaling system with embedded machine learning text depression detection alert.

[13] Trull TJ, Ebner-Priemer U (2013). Ambulatory assessment. Annu Rev Clin Psychol.

[14] Wu S, Yao M, Deng C, Marsiglia FF, Duan W (2021). Social isolation and anxiety disorder during the COVID-19 pandemic and lockdown in China. J Affect Disord.

[15] Liu J, Tai Z, Hu F (2024). Prevalence and coping of depression and anxiety among college students during COVID-19 lockdowns in China. J Affect Disord.

[16] Feng L, Zhang T, Wang Q (2021). Impact of COVID-19 outbreaks and interventions on influenza in China and the United States. Nat Commun.

[17] Wheaton MG, Abramowitz JS, Berman NC, Fabricant LE, Olatunji BO (2012). Psychological predictors of anxiety in response to the H1N1 (swine flu) pandemic. Cogn Ther Res.

[18] Ou Y, de Bruijn GJ, Schulz PJ (2025). Social media as an emotional barometer: bidirectional encoder representations from transformers-long short-term memory sentiment analysis on the evolution of public sentiments during influenza A on Sina Weibo. J Med Internet Res.

[19] Wang G, Sabran K (2024). Assessing depression and anxiety among young adults after epidemics and pandemics: a cross-sectional study in Anyang, China. Sci Rep.

[20] Bults M, Beaujean DJ, de Zwart O (2011). Perceived risk, anxiety, and behavioural responses of the general public during the early phase of the Influenza A (H1N1) pandemic in the Netherlands: results of three consecutive online surveys. BMC Public Health.

[21] Tausczik Y, Faasse K, Pennebaker JW, Petrie KJ (2012). Public anxiety and information seeking following the H1N1 outbreak: blogs, newspaper articles, and Wikipedia visits. Health Commun.

[22] Boiko DI, Skrypnikov AM, Shkodina AD, Hasan MM, Ashraf GM, Rahman MH (2022). Circadian rhythm disorder and anxiety as mental health complications in post-COVID-19. Environ Sci Pollut Res Int.

[23] Tian X, Yu G, He F (2016). An analysis of sleep complaints on Sina Weibo. Comput Human Behav.

[24] Tian X, He F, Batterham P, Wang Z, Yu G (2017). An analysis of anxiety-related postings on Sina Weibo. Int J Environ Res Public Health.

[25] Cox RC, Wright KP, Axelsson J, Balter LJT (2024). Diurnal variation in anxiety and activity is influenced by chronotype and probable anxiety-related disorder status. Psychiatry Res.

[26] Zou H, Bao C, Wang X (2025). Depressive symptoms moderate diurnal variation in response-inhibition-related neural activity among patients with depression: an EEG study. J Affect Disord.

[27] Taylor BJ, Hasler BP (2018). Chronotype and mental health: recent advances. Curr Psychiatry Rep.

[28] Ten Thij M, Bathina K, Rutter LA (2020). Depression alters the circadian pattern of online activity. Sci Rep.

[29] Substance Abuse and Mental Health Services Administration (2016). Impact of the DSM-IV to DSM-5 Changes on the National Survey on Drug Use and Health.

[30] (2025). Weekly report. Chinese National Influenza Center.

[31] Cao Y, Dai J, Wang Z (2025). Machine learning approaches for depression detection on social media: a systematic review of biases and methodological challenges. JBDS.

[32] Ali G, Mohd-Yasin F (2025). ARIMA modeling of noise of piezoelectric accelerometer. IEEE Access.

[33] Sabet SM, Dautovich ND, Dzierzewski JM (2021). The rhythm is gonna get you: social rhythms, sleep, depressive, and anxiety symptoms. J Affect Disord.

[34] Gleasure R, Saigot M, Kanat I (2024). Let’s talk about it in the morning: how circadian rhythms impact information sharing on social media. Affect Sci.

[35] Gleasure R, Davis FD, Riedl R, Brocke J, Léger PM, Randolph A, Fischer T (2020). Information Systems and Neuroscience: NeuroIS Retreat 2019 Lecture Notes in Information Systems and Organisation.

[36] Li Y, Luan S, Li Y, Hertwig R (2021). Changing emotions in the COVID-19 pandemic: a four-wave longitudinal study in the United States and China. Soc Sci Med.

[37] Li J, Luo C, Liu L (2024). Depression, anxiety, and insomnia symptoms among Chinese college students: a network analysis across pandemic stages. J Affect Disord.

[38] Metzler H, Rimé B, Pellert M, Niederkrotenthaler T, Di Natale A, Garcia D (2023). Collective emotions during the COVID-19 outbreak. Emotion.

[39] Ahmed A, Aziz S, Toro CT (2022). Machine learning models to detect anxiety and depression through social media: a scoping review. Comput Methods Programs Biomed Update.

[40] Eichstaedt JC, Schwartz HA, Kern ML (2015). Psychological language on Twitter predicts county-level heart disease mortality. Psychol Sci.

[41] Zhao Y, Cheng S, Yu X, Xu H (2020). Chinese public’s attention to the COVID-19 epidemic on social media: observational descriptive study. J Med Internet Res.

[42] Li X, Qing L, Wei H, Wei J (2026). Artificial intelligence and innovation ambidexterity in advanced manufacturing firms: a knowledge-management perspective. J Manuf Technol Manag.

[43] Ou Y, Zhang P, Yu J The application of the BERTopic model in natural language processing: in-depth text topic modeling.

[44] Ou Y, Sun F, Tan C, Shi H, Zhang M, Gong C Analyzing user behavior in online communities using data crawling and machine learning algorithms (erratum).

[45] Vannucci A, Flannery KM, Ohannessian CM (2017). Social media use and anxiety in emerging adults. J Affect Disord.

