# Supplementary material for: Temporal Dynamics of Influenza-Associated Anxiety Symptom Linguistic Markers on Weibo (2023-2024): Observational Study
Source: JMIR Infodemiology. 2026 Aug 7;6:e88849. doi: 10.2196/88849 (PMC13450889; doi:10.2196/88849)
Supplement: Supplementary file 1 [file infodemiology-v6-e88849-s001.zip › Multimedia Appendix 1/Attachment 3- Response Synonym Coverage.docx]

Precisely because of this high degree of linguistic variability, this study adopts a relatively conservative and concept-oriented approach in keyword selection. Specifically, rather than attempting to exhaustively include a large number of synonyms or colloquial expressions, this study selects only those Chinese translations that correspond most directly and clearly to the meanings of the English symptom terms. For example, based on the core symptom concepts defined in the diagnostic criteria for Generalized Anxiety Disorder (GAD), this study uses expressions such as “焦躁不安 / 感到紧张不安” (restlessness or feeling keyed up or on edge), “容易疲劳” (being easily fatigued), “注意力难以集中 / 脑子一片空白” (difficulty concentrating or mind going blank), “易怒” (irritability), “肌肉紧张” (muscle tension), and “睡眠障碍” (sleep disturbance) as retrieval keywords.

This keyword selection strategy is primarily based on two methodological considerations. First, using standardized expressions that directly correspond to clinical symptom descriptions helps maintain conceptual consistency between the retrieved texts and the theoretical framework of the study. Second, if a large number of semantically similar but conceptually unstable colloquial expressions were included in the retrieval process, the keyword set could expand rapidly and introduce a substantial amount of semantically ambiguous or weakly related content. This would increase data noise and reduce retrieval precision.

Therefore, the keyword system used in this study is centered on clinical symptom concepts and relies on their most direct Chinese equivalents as the basis for data retrieval, rather than constructing an ever-expanding network of synonyms. Although this approach may reduce coverage at the level of linguistic expression to some extent, it improves the precision of the correspondence between keywords and symptom concepts and helps ensure transparency, operational clarity, and replicability in the data collection process.

**To further assess the feasibility of exhaustively incorporating synonymous expressions, we conducted a pilot synonym-based scraping exercise following the committee’s suggestion. Specifically, for each symptom, we randomly selected 10 synonyms from a pool of approximately 200 expressions, resulting in a total of 80 keywords across 8 symptom categories. These keywords were then used to retrieve posts over 3 randomly selected days (January 1–3, 2024), on an hourly basis. After basic deduplication, this pilot yielded a total of 57,062 posts. This result indicates that even a limited subset of synonyms can already generate a substantial volume of data within a short time frame. Therefore, attempting to include all possible synonyms would likely lead to an extremely large and practically unmanageable dataset, which further supports our methodological choice.**

Following the committee’s suggestion, we have further clarified this methodological principle in the manuscript. Specifically, we state that the keywords used in this study are intended to represent the most direct linguistic expressions of the core symptom concepts rather than to exhaustively enumerate all possible semantic variants that may appear on Chinese social media.

In addition, to illustrate the richness and diversity of Chinese expressions, we provide in an appendix a list of potential synonymous expressions related to different anxiety symptoms. For each symptom category, approximately 200 related expressions are listed, including both relatively formal expressions and more colloquial forms commonly found in social media contexts. However, even with such an extensive list, it remains impossible to fully cover all possible semantic variants due to the complexity and openness of the Chinese language. We acknowledge this methodological limitation and have clarified it in the manuscript.

**Examples of Chinese expressions related to anxiety symptom concepts**

| Symptom Concept | Direct/relatively formal expressions | Colloquial / social media expressions |
| --- | --- | --- |
| Restlessness  / Feeling keyed up or on edge | Restlessness  1 焦躁不安 2 坐立不安 3 心神不宁 4 心绪不宁 5 心里不安 6 内心不安 7 情绪不安 8 精神不安 9 心情不安 10 心境不安  11 焦躁 12 情绪焦躁 13 内心焦躁 14 心情焦躁 15 心境焦躁 16 情绪烦躁 17 内心烦躁 18 心情烦躁 19 精神烦躁 20 情绪躁动  21 内心躁动 22 情绪不宁 23 内心不宁 24 心绪烦乱 25 心绪纷乱 26 思绪纷乱 27 情绪紊乱 28 心境紊乱 29 心境烦乱 30 心境躁动  31 精神紧张 32 精神紧绷 33 精神绷紧 34 神经紧张 35 神经紧绷 36 神经绷紧 37 精神处于紧张状态 38 精神处于紧绷状态 39 精神持续紧张 40 精神持续紧绷  41 心情难以平静 42 情绪难以平静 43 内心难以平静 44 心境难以平静 45 情绪难以安定 46 内心难以安定 47 心境难以安定 48 情绪难以稳定 49 内心难以稳定 50 心境难以稳定  51 情绪躁动不安 52 内心躁动不安 53 心境躁动不安 54 心情躁动不安 55 情绪烦躁不安 56 内心烦躁不安 57 心境烦躁不安 58 心情烦躁不安 59 情绪焦躁不安 60 内心焦躁不安  61 心境焦躁不安 62 心情焦躁不安 63 情绪波动不安 64 内心波动不安 65 心境波动不安 66 心情波动不安 67 情绪持续不安 68 内心持续不安 69 心境持续不安 70 心情持续不安  71 情绪持续烦躁 72 内心持续烦躁 73 心境持续烦躁 74 心情持续烦躁 75 情绪持续焦躁 76 内心持续焦躁 77 心境持续焦躁 78 心情持续焦躁 79 情绪持续躁动 80 内心持续躁动  81 心境持续躁动 82 心情持续躁动 83 情绪明显不安 84 内心明显不安 85 心境明显不安 86 心情明显不安 87 情绪明显烦躁 88 内心明显烦躁 89 心境明显烦躁 90 心情明显烦躁  91 情绪明显焦躁 92 内心明显焦躁 93 心境明显焦躁 94 心情明显焦躁 95 情绪明显躁动 96 内心明显躁动 97 心境明显躁动 98 心情明显躁动 99 情绪不安状态 100 内心不安状态  Feeling keyed up or on edge  1.精神紧张 2 精神紧绷 3 神经紧张 4 神经紧绷 5 情绪紧张 6 情绪紧绷 7 内心紧张 8 内心紧绷 9 心情紧张 10 心境紧张  11 心境紧绷 12 心神紧张 13 心神紧绷 14 精神绷紧 15 神经绷紧 16 情绪绷紧 17 心情绷紧 18 内心绷紧 19 心神绷紧 20 精神高度紧张  21 神经高度紧张 22 情绪高度紧张 23 内心高度紧张 24 心境高度紧张 25 心情高度紧张 26 精神处于紧张状态 27 神经处于紧张状态 28 情绪处于紧张状态 29 内心处于紧张状态 30 心境处于紧张状态  31 精神处于紧绷状态 32 神经处于紧绷状态 33 情绪处于紧绷状态 34 内心处于紧绷状态 35 心境处于紧绷状态 36 精神持续紧张 37 神经持续紧张 38 情绪持续紧张 39 内心持续紧张 40 心境持续紧张  41 精神持续紧绷 42 神经持续紧绷 43 情绪持续紧绷 44 内心持续紧绷 45 心境持续紧绷 46 精神保持紧张 47 神经保持紧张 48 情绪保持紧张 49 内心保持紧张 50 心境保持紧张  51 精神保持紧绷 52 神经保持紧绷 53 情绪保持紧绷 54 内心保持紧绷 55 心境保持紧绷 56 精神明显紧张 57 神经明显紧张 58 情绪明显紧张 59 内心明显紧张 60 心境明显紧张  61 精神明显紧绷 62 神经明显紧绷 63 情绪明显紧绷 64 内心明显紧绷 65 心境明显紧绷 66 精神持续绷紧 67 神经持续绷紧 68 情绪持续绷紧 69 内心持续绷紧 70 心境持续绷紧  71 精神处于警觉状态 72 神经处于警觉状态 73 情绪处于警觉状态 74 内心处于警觉状态 75 心境处于警觉状态 76 精神持续警觉 77 神经持续警觉 78 情绪持续警觉 79 内心持续警觉 80 心境持续警觉  81 精神高度警觉 82 神经高度警觉 83 情绪高度警觉 84 内心高度警觉 85 心境高度警觉 86 精神处于戒备状态 87 神经处于戒备状态 88 情绪处于戒备状态 89 内心处于戒备状态 90 心境处于戒备状态  91 精神持续戒备 92 神经持续戒备 93 情绪持续戒备 94 内心持续戒备 95 心境持续戒备 96 精神保持警觉 97 神经保持警觉 98 情绪保持警觉 99 内心保持警觉 100 心境保持警觉 | Restlessness  1 静不下来 2 坐不住 3 心里乱 4 心里乱糟糟 5 心里乱成一团 6 心里七上八下 7 心里没着没落 8 心里不踏实 9 心里慌 10 心里慌慌的  11 心里发慌 12 心里发躁 13 心里发烦 14 心里发乱 15 心里烦 16 心里烦得慌 17 心里躁 18 心里躁得慌 19 心里乱哄哄 20 心里不安稳  21 心里不安生 22 心里安不下来 23 心里静不下来 24 心里定不下来 25 心里定不住 26 心神不定 27 心神不安 28 心神不宁 29 心神乱 30 心神乱糟糟  31 心神慌 32 心神躁 33 心神不稳 34 心绪乱 35 心绪乱糟糟 36 心绪不宁 37 心绪不定 38 心绪烦 39 心绪躁 40 心绪慌  41 整个人很乱 42 整个人乱糟糟 43 整个人乱成一团 44 整个人不踏实 45 整个人安不下来 46 整个人静不下来 47 整个人定不住 48 整个人坐不住 49 整个人闲不住 50 整个人停不下来  51 整个人慌 52 整个人慌慌的 53 整个人烦 54 整个人烦得慌 55 整个人躁 56 整个人躁得慌 57 整个人乱哄哄 58 整个人不安稳 59 整个人不安生 60 整个人心里乱  61 人静不下来 62 人坐不住 63 人闲不住 64 人停不下来 65 人不踏实 66 人不安生 67 人不安稳 68 人心里乱 69 人心里慌 70 人心里烦  71 脑子乱 72 脑子乱糟糟 73 脑子乱成一团 74 脑子停不下来 75 脑子闲不住 76 脑子定不下来 77 脑子定不住 78 脑子慌 79 脑子烦 80 脑子躁  81 心里像打鼓 82 心里像揣着东西 83 心里像悬着 84 心里像堵着 85 心里像压着 86 心里像翻腾 87 心里像乱线 88 心里像一团麻 89 心里像乱麻 90 心里像开锅  91 心里翻腾 92 心里折腾 93 心里闹腾 94 心里发紧 95 心里揪着 96 心里提着 97 心里悬着 98 心里绷着 99 心里堵着 100 心里乱着  Feeling keyed up or on edge  1 整个人很紧 2 整个人很紧绷 3 整个人绷着 4 整个人绷得很紧 5 整个人一直绷着 6 整个人紧着 7 整个人紧绷着 8 整个人紧着劲 9 整个人绷着劲 10 整个人紧得很  11 身体很紧 12 身体紧绷 13 身体绷着 14 身体绷得很紧 15 身体一直绷着 16 身体紧着 17 身体紧绷着 18 身体紧着劲 19 身体绷着劲 20 身体紧得很  21 心里紧 22 心里绷着 23 心里绷得很紧 24 心里紧绷 25 心里紧着 26 心里紧着劲 27 心里绷着劲 28 心里紧得很 29 心里一直绷着 30 心里一直紧着  31 心一直提着 32 心里提着 33 心里提着一口气 34 心里一直提着 35 心一直悬着 36 心里悬着 37 心里悬着一口气 38 心里吊着 39 心里一直吊着 40 心一直吊着  41 心里绷得慌 42 心里紧得慌 43 心里绷着不放 44 心里紧着不放 45 心里一直紧着 46 心里一直绷着 47 心里绷得厉害 48 心里紧得厉害 49 心里绷着不松 50 心里紧着不松  51 整个人很绷 52 整个人绷得慌 53 整个人紧得慌 54 整个人绷得厉害 55 整个人紧得厉害 56 整个人绷着不放 57 整个人紧着不放 58 整个人绷着不松 59 整个人紧着不松 60 整个人紧绷得很  61 身体绷得慌 62 身体紧得慌 63 身体绷得厉害 64 身体紧得厉害 65 身体绷着不放 66 身体紧着不放 67 身体绷着不松 68 身体紧着不松 69 身体紧绷得很 70 身体绷得很紧  71 心里像绷着一根弦 72 整个人像绷着一根弦 73 身体像绷着一根弦 74 心里像拉紧的弦 75 整个人像拉紧的弦 76 身体像拉紧的弦 77 心里像绷紧 78 整个人像绷紧 79 身体像绷紧 80 心里像拉紧  81 心里紧着一根弦 82 整个人紧着一根弦 83 身体紧着一根弦 84 心里一直紧着弦 85 整个人一直紧着弦 86 身体一直紧着弦 87 心里绷着弦 88 整个人绷着弦 89 身体绷着弦 90 心里一直绷着弦  91 心里紧张得很 92 心里紧张得慌 93 心里紧张得厉害 94 心里紧张不放 95 心里紧张不松 96 整个人紧张得很 97 整个人紧张得慌 98 身体紧张得很 99 身体紧张得慌 100 整个人紧张得厉害 |
| Being easily fatigued | 1 容易疲劳 2 容易感到疲劳 3 易感疲劳 4 容易疲惫 5 容易感到疲惫 6 易感疲惫 7 容易劳累 8 容易感到劳累 9 易感劳累 10 容易乏力 11 容易感到乏力 12 易感乏力 13 容易体力不支 14 容易精力不足 15 精力容易耗尽 16 容易精疲力尽 17 容易身心疲惫 18 容易身心疲劳 19 容易体力透支 20 容易出现疲劳感 21 容易出现疲惫感 22 容易产生疲劳感 23 容易产生疲惫感 24 疲劳感明显 25 疲惫感明显 26 疲劳感较强 27 疲惫感较强 28 持续疲劳 29 持续疲惫 30 长期疲劳 31 长期疲惫 32 慢性疲劳 33 慢性疲惫 34 身体疲劳明显 35 身体疲惫明显 36 身体容易疲劳 37 身体容易疲惫 38 精神容易疲劳 39 精神容易疲惫 40 精神疲劳明显 41 精神疲惫明显 42 精力明显下降 43 精力明显不足 44 精力持续不足 45 精力持续下降 46 精力难以维持 47 精力难以恢复 48 精力消耗较快 49 精力恢复缓慢 50 精力容易耗尽 51 体力明显下降 52 体力明显不足 53 体力持续不足 54 体力持续下降 55 体力恢复缓慢 56 体力容易耗尽 57 体力难以恢复 58 体力消耗较快 59 容易体力下降 60 容易体力不足 61 身体持续疲劳 62 身体持续疲惫 63 身体长期疲劳 64 身体长期疲惫 65 精神持续疲劳 66 精神持续疲惫 67 精神长期疲劳 68 精神长期疲惫 69 精神状态疲惫 70 身体状态疲惫 71 身体状态疲劳 72 精神状态疲劳 73 身心持续疲劳 74 身心持续疲惫 75 身心长期疲劳 76 身心长期疲惫 77 身体容易劳累 78 精神容易劳累 79 精力恢复困难 80 体力恢复困难 81 精力持续消耗 82 体力持续消耗 83 精力明显消耗 84 体力明显消耗 85 精神状态低落疲惫 86 身体状态低落疲惫 87 身体持续乏力 88 精神持续乏力 89 身体明显乏力 90 精神明显乏力 91 精力明显衰减 92 体力明显衰减 93 精神明显衰减 94 身体明显衰减 95 精力下降明显 96 体力下降明显 97 身体疲劳持续 98 精神疲劳持续 99 身体疲惫持续 100 精神疲惫持续 | 1 好累 2 很累 3 特别累 4 累得不行 5 累到不行 6 累死了 7 累惨了 8 累坏了 9 累瘫了 10 累垮了 11 累爆了 12 累到爆 13 累到虚脱 14 累到崩溃 15 累到没力气 16 累得没劲 17 累得动不了 18 累得不想动 19 累得不想说话 20 累得不想出门 21 累得不想起床 22 累到想躺平 23 累得整个人软掉 24 累得整个人散架 25 累得整个人没电 26 累得整个人空了 27 累得整个人废了 28 累得整个人虚了 29 累得整个人垮了 30 累得整个人不行 31 累到没精神 32 累到没精力 33 累到没状态 34 累到不想动脑 35 累到脑子不转 36 累到脑子空空 37 累到脑子不动 38 累到脑袋发懵 39 累到头都大了 40 累到头昏 41 累到头晕 42 累到不想干活 43 累到不想学习 44 累到不想工作 45 累到整个人蔫了 46 累到整个人没劲 47 累到整个人没电 48 累到整个人垮掉 49 累到整个人瘫 50 累到整个人散架 51 整个人好累 52 整个人特别累 53 整个人累惨了 54 整个人累到不行 55 整个人累得慌 56 整个人累坏了 57 整个人累瘫 58 整个人累垮 59 整个人没劲 60 整个人没精神 61 整个人没精力 62 整个人没状态 63 整个人没电 64 整个人虚掉 65 整个人蔫了 66 整个人疲惫 67 整个人疲得不行 68 整个人累得慌 69 整个人累炸了 70 整个人累爆了 71 整个人累到爆 72 整个人累到虚脱 73 整个人累到崩溃 74 整个人累到不想动 75 整个人累到不想说话 76 整个人累到不想出门 77 整个人累到不想起床 78 整个人累到想躺 79 整个人累到只想睡 80 整个人累到想摆烂 81 整个人累得发懵 82 整个人累得发虚 83 整个人累得发软 84 整个人累得发蔫 85 整个人累得发空 86 整个人累得发散 87 整个人累得不行 88 整个人累到极限 89 整个人累到极点 90 整个人累到顶 91 整个人累到头 92 整个人累到爆表 93 整个人累到极度 94 整个人累到极限状态 95 整个人累到撑不住 96 整个人累到顶不住 97 整个人累到不行了 98 整个人累到崩 99 整个人累到没力 100 整个人累到没劲 |
| Difficulty concentrating  or mind going blank | Difficulty concentrating  1 注意力难以集中 2 难以集中注意力 3 注意力不集中 4 注意力难以保持 5 难以维持注意力 6 注意力容易分散 7 注意力容易涣散 8 注意力持续困难 9 注意力保持困难 10 注意力维持困难 11 专注困难 12 难以专注 13 难以保持专注 14 专注能力下降 15 专注能力不足 16 专注能力减弱 17 专注能力受损 18 专注能力降低 19 专注能力下降明显 20 专注能力难以维持 21 思维难以集中 22 思绪难以集中 23 思维分散 24 思绪分散 25 思维涣散 26 思绪涣散 27 思维难以维持 28 思绪难以维持 29 思维难以持续 30 思绪难以持续 31 思维不集中 32 思绪不集中 33 思维难以聚焦 34 思绪难以聚焦 35 思维难以整理 36 思绪难以整理 37 思维混乱 38 思绪混乱 39 思维紊乱 40 思绪紊乱 41 思维难以清晰 42 思绪难以清晰 43 思维难以组织 44 思绪难以组织 45 思维难以协调 46 思绪难以协调 47 思维难以控制 48 思绪难以控制 49 思维难以稳定 50 思绪难以稳定 51 思维难以保持稳定 52 思绪难以保持稳定 53 思维连贯性下降 54 思绪连贯性下降 55 思维连贯性不足 56 思绪连贯性不足 57 思维连贯性受损 58 思绪连贯性受损 59 思维效率下降 60 思维效率明显下降 61 思维运转困难 62 思维运转不畅 63 思维运转缓慢 64 思维运转受阻 65 思维处理困难 66 信息处理困难 67 信息处理能力下降 68 信息处理能力受损 69 信息处理能力不足 70 思维整合困难 71 思维整合能力下降 72 思维整合能力受损 73 思维组织困难 74 思维整理困难 75 思维协调困难 76 思绪协调困难 77 思维聚焦困难 78 思绪聚焦困难 79 思维持续能力下降 80 思绪持续能力下降 81 思维稳定性下降 82 思绪稳定性下降 83 思维稳定性不足 84 思绪稳定性不足 85 思维控制能力下降 86 思绪控制能力下降 87 思维调节能力下降 88 思绪调节能力下降 89 思维清晰度下降 90 思绪清晰度下降 91 思维清晰度不足 92 思绪清晰度不足 93 思维清晰度受损 94 思绪清晰度受损 95 思维难以集中于任务 96 思维难以集中于活动 97 注意力控制困难 98 注意力调节困难 99 注意力管理困难 100 注意力分配困难  Mind going blank  1 思维空白 2 思维突然空白 3 思维短暂空白 4 思维暂时空白 5 思维出现空白 6 思维突然停顿 7 思维突然中断 8 思维短暂停顿 9 思维短暂中断 10 思维暂时停顿 11 思维暂时中断 12 思维短暂停滞 13 思维突然停滞 14 思维暂时停滞 15 思维瞬间停滞 16 思维突然停止 17 思维短暂停止 18 思维暂时停止 19 思维突然失去连贯性 20 思维短暂失去连贯性 21 思维暂时失去连贯性 22 思维瞬间断裂 23 思维短暂断裂 24 思维暂时断裂 25 思维突然缺失 26 思维短暂缺失 27 思维暂时缺失 28 思维突然失去 29 思维短暂失去 30 思维暂时失去 31 思绪空白 32 思绪突然空白 33 思绪短暂空白 34 思绪暂时空白 35 思绪出现空白 36 思绪突然停顿 37 思绪突然中断 38 思绪短暂停顿 39 思绪短暂中断 40 思绪暂时停顿 41 思绪暂时中断 42 思绪短暂停滞 43 思绪突然停滞 44 思绪暂时停滞 45 思绪瞬间停滞 46 思绪突然停止 47 思绪短暂停止 48 思绪暂时停止 49 思绪突然失去连贯性 50 思绪短暂失去连贯性 51 思绪暂时失去连贯性 52 思绪瞬间断裂 53 思绪短暂断裂 54 思绪暂时断裂 55 思绪突然缺失 56 思绪短暂缺失 57 思绪暂时缺失 58 思绪突然失去 59 思绪短暂失去 60 思绪暂时失去 61 思维突然模糊 62 思维短暂模糊 63 思维暂时模糊 64 思维瞬间模糊 65 思维突然混乱 66 思维短暂混乱 67 思维暂时混乱 68 思维瞬间混乱 69 思绪突然模糊 70 思绪短暂模糊 71 思绪暂时模糊 72 思绪瞬间模糊 73 思绪突然混乱 74 思绪短暂混乱 75 思绪暂时混乱 76 思绪瞬间混乱 77 思维短暂失去清晰度 78 思维暂时失去清晰度 79 思维突然失去清晰度 80 思维短暂失去思考能力 81 思维暂时失去思考能力 82 思维突然失去思考能力 83 思绪短暂失去思考能力 84 思绪暂时失去思考能力 85 思绪突然失去思考能力 86 思维突然停止运转 87 思维短暂停止运转 88 思维暂时停止运转 89 思绪突然停止运转 90 思绪短暂停止运转 91 思绪暂时停止运转 92 思维突然无法继续 93 思维短暂无法继续 94 思维暂时无法继续 95 思绪突然无法继续 96 思绪短暂无法继续 97 思绪暂时无法继续 98 思维短暂失去内容 99 思维暂时失去内容 100 思维突然失去内容 | Difficulty concentrating  1 注意力老是跑 2 注意力老是散 3 注意力老是飘 4 注意力不在 5 注意力不在线 6 注意力跟不上 7 注意力总是跑掉 8 注意力很难集中 9 注意力特别难集中 10 注意力老是集中不了 11 脑子不集中 12 脑子很难集中 13 脑子集中不了 14 脑子老是跑神 15 脑子老是走神 16 老是走神 17 经常走神 18 总是走神 19 老是分心 20 很容易分心 21 动不动就分心 22 总是分心 23 老是心不在焉 24 一直心不在焉 25 老是发呆 26 动不动发呆 27 经常发呆 28 脑子老是飘 29 脑子老是乱跑 30 脑子不在状态 31 脑子不在线 32 脑子跟不上 33 脑子慢半拍 34 脑子反应慢 35 脑子转不过来 36 脑子老是乱 37 脑子很乱 38 脑子乱糟糟 39 脑子乱七八糟 40 脑子理不清 41 脑子理不顺 42 脑子想不明白 43 脑子想不出来 44 脑子不太好使 45 脑子不好使 46 脑子不太灵 47 脑子不灵 48 脑子有点卡 49 脑子有点卡壳 50 脑子有点卡住 51 整个人注意力不集中 52 整个人老是走神 53 整个人老是分心 54 整个人心不在焉 55 整个人注意力飘 56 整个人注意力散 57 整个人脑子乱 58 整个人脑子飘 59 整个人脑子跟不上 60 整个人脑子慢半拍 61 整个人脑子不在线 62 整个人脑子不集中 63 整个人脑子很乱 64 整个人脑子理不清 65 整个人脑子理不顺 66 整个人脑子想不明白 67 整个人脑子想不出来 68 整个人脑子不好使 69 整个人脑子不太灵 70 整个人脑子卡住 71 整个人脑子卡壳 72 整个人脑子反应慢 73 整个人脑子乱糟糟 74 整个人脑子乱七八糟 75 整个人脑子不在状态 76 整个人注意力不在 77 整个人注意力跑掉 78 整个人注意力飘走 79 整个人注意力老是散 80 整个人注意力老是跑 81 整个人注意力很难集中 82 整个人注意力集中不了 83 整个人很容易分心 84 整个人老是发呆 85 整个人动不动发呆 86 整个人老是走神 87 整个人经常走神 88 整个人总是走神 89 整个人注意力不稳 90 整个人注意力不太集中 91 整个人注意力很散 92 整个人注意力很飘 93 整个人注意力很乱 94 整个人注意力理不清 95 整个人注意力理不顺 96 整个人注意力跟不上 97 整个人注意力慢半拍 98 整个人注意力不在线 99 整个人注意力不在状态 100 整个人注意力难集中  Mind going blank  1 脑子一片空白 2 脑子空白 3 脑子突然空白 4 脑子一下空白 5 脑子突然空了 6 脑子空空 7 脑子突然空空 8 脑子空掉了 9 脑子突然断片 10 脑子断片 11 脑子突然卡住 12 脑子卡住 13 脑子卡壳 14 脑子突然卡壳 15 脑子短路 16 脑子突然短路 17 脑子死机 18 脑子突然死机 19 脑子宕机 20 脑子突然宕机 21 脑子停摆 22 脑子突然停摆 23 脑子停机 24 脑子突然停机 25 脑子当机 26 脑子突然当机 27 脑子不转 28 脑子突然不转 29 脑子转不动 30 脑子突然转不动 31 脑子发懵 32 脑子突然发懵 33 脑子懵了 34 脑子突然懵了 35 脑子一下懵 36 脑子一时懵 37 脑子有点懵 38 脑子很懵 39 脑子特别懵 40 脑子懵懵的 41 脑子一下断线 42 脑子掉线 43 脑子突然掉线 44 脑子断线 45 脑子突然断线 46 脑子不在线 47 脑子突然不在线 48 脑子不在状态 49 脑子突然不在状态 50 脑子突然想不起来 51 脑子想不起来 52 脑子一时想不起 53 脑子一时想不起来 54 脑子突然忘了 55 脑子突然一片空 56 脑子一下卡住 57 脑子一下短路 58 脑子一下死机 59 脑子一下宕机 60 脑子一下停摆 61 脑子一下停机 62 整个人脑子空白 63 整个人脑子空空 64 整个人脑子断片 65 整个人脑子卡住 66 整个人脑子卡壳 67 整个人脑子短路 68 整个人脑子死机 69 整个人脑子宕机 70 整个人脑子停摆 71 整个人脑子停机 72 整个人脑子不转 73 整个人脑子转不动 74 整个人脑子发懵 75 整个人脑子懵了 76 整个人脑子掉线 77 整个人脑子断线 78 整个人脑子不在线 79 整个人脑子不在状态 80 整个人脑子想不起来 81 整个人脑子突然空白 82 整个人脑子突然卡住 83 整个人脑子突然短路 84 整个人脑子突然死机 85 整个人脑子突然宕机 86 整个人脑子突然停摆 87 整个人脑子突然停机 88 整个人脑子突然不转 89 整个人脑子突然懵 90 整个人脑子突然掉线 91 整个人脑子突然断线 92 整个人脑子突然不在线 93 整个人脑子突然不在状态 94 整个人脑子突然想不起来 95 整个人脑子突然断片 96 整个人脑子突然卡壳 97 整个人脑子一下空 98 整个人脑子一下懵 99 整个人脑子一下断线 100 整个人脑子一下短路 |
| Irritability | 1 易怒 2 容易生气 3 容易发怒 4 容易发火 5 容易恼怒 6 容易烦躁 7 容易不耐烦 8 易被激怒 9 容易被激怒 10 容易被惹怒 11 情绪易怒 12 情绪容易生气 13 情绪容易发怒 14 情绪容易发火 15 情绪容易恼怒 16 情绪容易烦躁 17 情绪容易不耐烦 18 情绪易被激怒 19 情绪容易被激怒 20 情绪容易被惹怒 21 比较容易生气 22 比较容易发怒 23 比较容易发火 24 比较容易恼怒 25 比较容易烦躁 26 比较容易不耐烦 27 比较容易被激怒 28 比较容易被惹怒 29 相对容易生气 30 相对容易发怒 31 很容易生气 32 很容易发怒 33 很容易发火 34 很容易恼怒 35 很容易烦躁 36 很容易不耐烦 37 很容易被激怒 38 很容易被惹怒 39 特别容易生气 40 特别容易发怒 41 特别容易发火 42 特别容易恼怒 43 特别容易烦躁 44 特别容易不耐烦 45 特别容易被激怒 46 特别容易被惹怒 47 非常容易生气 48 非常容易发怒 49 非常容易发火 50 非常容易恼怒 51 非常容易烦躁 52 非常容易不耐烦 53 非常容易被激怒 54 非常容易被惹怒 55 情绪比较容易生气 56 情绪比较容易发怒 57 情绪比较容易发火 58 情绪比较容易恼怒 59 情绪比较容易烦躁 60 情绪比较容易不耐烦 61 情绪很容易生气 62 情绪很容易发怒 63 情绪很容易发火 64 情绪很容易恼怒 65 情绪很容易烦躁 66 情绪很容易不耐烦 67 情绪很容易被激怒 68 情绪很容易被惹怒 69 情绪特别容易生气 70 情绪特别容易发怒 71 情绪特别容易发火 72 情绪特别容易恼怒 73 情绪特别容易烦躁 74 情绪特别容易不耐烦 75 情绪特别容易被激怒 76 情绪特别容易被惹怒 77 情绪非常容易生气 78 情绪非常容易发怒 79 情绪非常容易发火 80 情绪非常容易恼怒 81 情绪非常容易烦躁 82 情绪非常容易不耐烦 83 情绪非常容易被激怒 84 情绪非常容易被惹怒 85 对小事容易生气 86 对小事容易发怒 87 对小事容易发火 88 对小事容易恼怒 89 对小事容易烦躁 90 对小事容易不耐烦 91 对小事很容易生气 92 对小事很容易发怒 93 对小事很容易发火 94 对小事很容易恼怒 95 对小事很容易烦躁 96 对小事很容易不耐烦 97 对小事特别容易生气 98 对小事特别容易发怒 99 对小事特别容易发火 100 对小事特别容易烦躁 | 1 很容易生气 2 动不动就生气 3 老是生气 4 总是生气 5 一点就生气 6 一点点就生气 7 一点小事就生气 8 很容易发火 9 动不动就发火 10 老是发火 11 总是发火 12 一点就发火 13 一点小事就发火 14 很容易炸 15 动不动就炸 16 一点就炸 17 一点点就炸 18 一点小事就炸 19 很容易炸毛 20 动不动就炸毛 21 一点就炸毛 22 老是炸毛 23 总是炸毛 24 脾气很大 25 脾气特别大 26 脾气越来越大 27 脾气变大了 28 脾气变差了 29 脾气不好 30 脾气很差 31 脾气很冲 32 脾气很急 33 脾气很暴 34 脾气很躁 35 脾气很火 36 脾气有点爆 37 脾气容易爆 38 脾气容易炸 39 脾气容易发火 40 脾气容易生气 41 火气很大 42 火气特别大 43 火气很重 44 火气很旺 45 火气上来很快 46 火气一上来就爆 47 火气一上来就炸 48 动不动就来气 49 老是来气 50 总是来气 51 很容易烦 52 动不动就烦 53 老是烦 54 总是烦 55 很容易烦躁 56 动不动就烦躁 57 老是烦躁 58 总是烦躁 59 很容易不耐烦 60 动不动就不耐烦 61 老是不耐烦 62 总是不耐烦 63 一点就不耐烦 64 一点小事就不耐烦 65 情绪很容易爆 66 情绪动不动就爆 67 情绪一上来就爆 68 情绪一上来就炸 69 情绪很冲 70 情绪很躁 71 情绪很烦 72 情绪很急 73 情绪很火 74 情绪很炸 75 情绪很暴 76 情绪很怒 77 情绪一上来就火 78 情绪一上来就怒 79 情绪一上来就冲 80 情绪一上来就躁 81 整个人很暴躁 82 整个人很烦躁 83 整个人火气很大 84 整个人脾气很大 85 整个人容易发火 86 整个人容易生气 87 整个人很容易炸 88 整个人动不动就炸 89 整个人一点就炸 90 整个人很不耐烦 91 整个人动不动就不耐烦 92 整个人老是烦 93 整个人老是暴躁 94 整个人脾气很冲 95 整个人脾气很急 96 整个人脾气很躁 97 整个人火气很冲 98 整个人情绪很炸 99 整个人情绪很冲 100 整个人情绪很暴 |
| Muscle tension | 1 肌肉紧张 2 肌肉很紧张 3 肌肉有些紧张 4 肌肉比较紧张 5 肌肉持续紧张 6 肌肉一直紧张 7 肌肉明显紧张 8 肌肉特别紧张 9 肌肉非常紧张 10 肌肉长期紧张  11 肌肉处于紧张状态 12 肌肉保持紧张 13 肌肉容易紧张 14 肌肉变得紧张 15 肌肉逐渐紧张 16 肌肉开始紧张 17 肌肉变得很紧张 18 肌肉持续处于紧张 19 肌肉一直处于紧张 20 肌肉处于持续紧张  21 肌肉紧绷 22 肌肉很紧绷 23 肌肉有些紧绷 24 肌肉比较紧绷 25 肌肉持续紧绷 26 肌肉一直紧绷 27 肌肉明显紧绷 28 肌肉特别紧绷 29 肌肉非常紧绷 30 肌肉长期紧绷  31 肌肉处于紧绷状态 32 肌肉保持紧绷 33 肌肉容易紧绷 34 肌肉变得紧绷 35 肌肉逐渐紧绷 36 肌肉开始紧绷 37 肌肉变得很紧绷 38 肌肉持续处于紧绷 39 肌肉一直处于紧绷 40 肌肉处于持续紧绷  41 身体紧张 42 身体很紧张 43 身体有些紧张 44 身体比较紧张 45 身体持续紧张 46 身体一直紧张 47 身体明显紧张 48 身体特别紧张 49 身体非常紧张 50 身体长期紧张  51 身体处于紧张状态 52 身体保持紧张 53 身体容易紧张 54 身体变得紧张 55 身体逐渐紧张 56 身体开始紧张 57 身体变得很紧张 58 身体持续处于紧张 59 身体一直处于紧张 60 身体处于持续紧张  61 身体紧绷 62 身体很紧绷 63 身体有些紧绷 64 身体比较紧绷 65 身体持续紧绷 66 身体一直紧绷 67 身体明显紧绷 68 身体特别紧绷 69 身体非常紧绷 70 身体长期紧绷  71 身体处于紧绷状态 72 身体保持紧绷 73 身体容易紧绷 74 身体变得紧绷 75 身体逐渐紧绷 76 身体开始紧绷 77 身体变得很紧绷 78 身体持续处于紧绷 79 身体一直处于紧绷 80 身体处于持续紧绷  81 肌肉难以放松 82 肌肉很难放松 83 肌肉不易放松 84 肌肉难以完全放松 85 肌肉一直难以放松 86 肌肉持续难以放松 87 肌肉明显难以放松 88 肌肉比较难放松 89 肌肉非常难放松 90 肌肉长期难以放松  91 身体难以放松 92 身体很难放松 93 身体不易放松 94 身体难以完全放松 95 身体一直难以放松 96 身体持续难以放松 97 身体明显难以放松 98 身体比较难放松 99 身体非常难放松 100 身体长期难以放松 | 1 身体很紧 2 身体特别紧 3 身体有点紧 4 身体一直很紧 5 身体老是很紧 6 身体总是很紧 7 身体变得很紧 8 身体越来越紧 9 身体紧得很 10 身体紧得不行  11 身体紧绷 12 身体很紧绷 13 身体特别紧绷 14 身体有点紧绷 15 身体一直紧绷 16 身体老是紧绷 17 身体总是紧绷 18 身体变得紧绷 19 身体越来越紧绷 20 身体紧绷得很  21 身体绷着 22 身体一直绷着 23 身体老是绷着 24 身体总是绷着 25 身体绷得很紧 26 身体绷得很厉害 27 身体绷得不行 28 身体绷得很明显 29 身体绷着不放松 30 身体绷着不松  31 身体很僵 32 身体特别僵 33 身体有点僵 34 身体一直很僵 35 身体老是很僵 36 身体总是很僵 37 身体变得很僵 38 身体越来越僵 39 身体僵得很 40 身体僵得不行  41 身体僵硬 42 身体很僵硬 43 身体特别僵硬 44 身体有点僵硬 45 身体一直僵硬 46 身体老是僵硬 47 身体总是僵硬 48 身体变得僵硬 49 身体越来越僵硬 50 身体僵硬得很  51 身体放松不了 52 身体一直放松不了 53 身体老是放松不了 54 身体总是放松不了 55 身体就是放松不了 56 身体完全放松不了 57 身体很难放松 58 身体特别难放松 59 身体一直很难放松 60 身体老是很难放松  61 身体紧着 62 身体一直紧着 63 身体老是紧着 64 身体总是紧着 65 身体紧着不放 66 身体紧着不松 67 身体紧着很久 68 身体紧着不舒服 69 身体紧着难受 70 身体紧着不太对劲  71 整个人很紧 72 整个人特别紧 73 整个人有点紧 74 整个人一直很紧 75 整个人老是很紧 76 整个人总是很紧 77 整个人变得很紧 78 整个人越来越紧 79 整个人紧得很 80 整个人紧得不行  81 整个人紧绷 82 整个人很紧绷 83 整个人特别紧绷 84 整个人有点紧绷 85 整个人一直紧绷 86 整个人老是紧绷 87 整个人总是紧绷 88 整个人变得紧绷 89 整个人越来越紧绷 90 整个人紧绷得很  91 整个人绷着 92 整个人一直绷着 93 整个人老是绷着 94 整个人总是绷着 95 整个人绷得很紧 96 整个人绷得很厉害 97 整个人绷得不行 98 整个人绷着不放松 99 整个人绷着不松 100 整个人一直绷着劲 |
| Sleep disturbance | 1 睡眠障碍 2 睡眠问题 3 睡眠困难 4 睡眠状况不佳 5 睡眠质量差 6 睡眠质量不好 7 睡眠质量下降 8 睡眠质量变差 9 睡眠状态不好 10 睡眠状态不佳  11 睡眠状况变差 12 睡眠状况下降 13 睡眠不良 14 睡眠不佳 15 睡眠质量持续下降 16 睡眠质量持续变差 17 睡眠质量明显下降 18 睡眠质量明显变差 19 睡眠持续不佳 20 睡眠持续不好  21 睡眠持续变差 22 睡眠持续下降 23 睡眠问题明显 24 睡眠问题持续 25 睡眠问题加重 26 睡眠问题增加 27 睡眠问题严重 28 睡眠问题明显增加 29 睡眠问题逐渐加重 30 睡眠问题逐渐增加  31 失眠 32 持续失眠 33 长期失眠 34 经常失眠 35 反复失眠 36 偶尔失眠 37 间歇性失眠 38 短期失眠 39 慢性失眠 40 明显失眠  41 入睡困难 42 难以入睡 43 入睡困难明显 44 入睡困难增加 45 入睡困难持续 46 入睡困难加重 47 入睡困难明显增加 48 入睡困难逐渐加重 49 入睡困难持续存在 50 入睡困难较为明显  51 睡眠不稳定 52 睡眠不规律 53 睡眠质量不稳定 54 睡眠状态不稳定 55 睡眠时间不稳定 56 睡眠持续不稳定 57 睡眠明显不稳定 58 睡眠越来越差 59 睡眠逐渐变差 60 睡眠逐渐下降  61 睡眠持续下降 62 睡眠持续变差 63 睡眠明显变差 64 睡眠明显下降 65 睡眠情况变差 66 睡眠情况下降 67 睡眠情况不好 68 睡眠情况不佳 69 睡眠持续不好 70 睡眠持续不佳  71 夜间易醒 72 夜间容易醒来 73 夜间醒来频繁 74 夜间反复醒来 75 夜间睡眠中断 76 夜间睡眠不稳 77 夜间睡眠不佳 78 夜间睡眠质量差 79 夜间睡眠质量不好 80 夜间睡眠质量下降  81 夜间睡眠变差 82 夜间睡眠持续变差 83 夜间睡眠持续下降 84 夜间睡眠明显下降 85 夜间睡眠明显变差 86 夜间睡眠不稳定 87 夜间睡眠状态不好 88 夜间睡眠状态不佳 89 夜间睡眠问题 90 夜间睡眠障碍  91 睡眠困难明显增加 92 睡眠困难持续存在 93 睡眠困难逐渐加重 94 睡眠困难逐渐增加 95 睡眠困难越来越明显 96 睡眠问题越来越明显 97 睡眠问题越来越严重 98 睡眠状态持续不好 99 睡眠状态持续不佳 100 睡眠问题持续存在 | 1 睡不好 2 最近睡不好 3 一直睡不好 4 总是睡不好 5 老是睡不好 6 最近一直睡不好 7 最近老是睡不好 8 最近总是睡不好 9 睡得不好 10 睡得不太好  11 睡得不怎么样 12 睡得不行 13 睡得不太行 14 睡得不太安稳 15 睡得不踏实 16 睡得很差 17 睡得特别差 18 睡得越来越差 19 睡得越来越不好 20 睡得明显不好  21 睡不着 22 老是睡不着 23 总是睡不着 24 一直睡不着 25 最近睡不着 26 最近老是睡不着 27 最近一直睡不着 28 最近总是睡不着 29 怎么都睡不着 30 一直很难睡着  31 很难睡着 32 特别难睡着 33 最近很难睡着 34 最近特别难睡着 35 最近一直很难睡着 36 最近老是很难睡着 37 最近总是很难睡着 38 睡得很晚 39 总是睡得很晚 40 最近睡得很晚  41 老是睡得很晚 42 总是睡得特别晚 43 最近总是睡得很晚 44 最近老是睡得很晚 45 最近一直睡得很晚 46 半夜醒 47 老是半夜醒 48 总是半夜醒 49 最近老是半夜醒 50 最近总是半夜醒  51 半夜老醒 52 半夜总醒 53 夜里老醒 54 夜里总醒 55 最近夜里老醒 56 最近夜里总醒 57 夜里醒很多次 58 夜里醒好几次 59 夜里反复醒 60 夜里老是醒  61 夜里总是醒 62 最近夜里老是醒 63 最近夜里总是醒 64 睡得不踏实 65 最近睡得不踏实 66 老是睡得不踏实 67 总是睡得不踏实 68 最近总是睡得不踏实 69 最近老是睡得不踏实 70 最近一直睡得不踏实  71 睡眠很差 72 最近睡眠很差 73 最近睡眠特别差 74 最近睡眠越来越差 75 最近睡眠越来越不好 76 最近睡眠不太好 77 最近睡眠不怎么样 78 最近睡眠不太行 79 最近睡眠很不好 80 最近睡眠明显不好  81 最近睡觉不好 82 最近睡觉很差 83 最近睡觉特别差 84 最近睡觉越来越差 85 最近睡觉不太好 86 最近睡觉不太行 87 最近睡觉不怎么样 88 最近睡觉很不好 89 最近睡觉明显不好 90 最近睡觉特别不好  91 最近总是睡不好 92 最近一直睡不好 93 最近老是睡不好 94 最近怎么都睡不好 95 最近一直睡不着 96 最近老是睡不着 97 最近总是睡不着 98 最近怎么都睡不着 99 最近睡得特别不好 100 最近睡得越来越不好 |

NOTE: *The expressions listed above are illustrative rather than exhaustive. In practice, Chinese social media users may employ many additional colloquial or metaphorical expressions to describe similar psychological states.*
