# Supplementary material for: Temporal Dynamics of Influenza-Associated Anxiety Symptom Linguistic Markers on Weibo (2023-2024): Observational Study
Source: JMIR Infodemiology. 2026 Aug 7;6:e88849. doi: 10.2196/88849 (PMC13450889; doi:10.2196/88849)
Supplement: Supplementary file 5 [file infodemiology-v6-e88849-s002.doc]

**Data Preprocessing**

To better understand the textual features and thematic structures of those posts, we utilized Python’s “re” module, which handles regular expressions, to eliminate all non-Chinese elements from the data in the following iterative manner:

(1)Remove specific emojis.

(2) Replace Unicode special characters: Match and replace characters outside the Basic Multilingual Plane (e.g., [\U00010000-\U0010ffff] or surrogate pairs [\uD800-\uDBFF][\uDC00-\uDFFF]), which will be replaced by restr (default is an empty string, i.e., removal).

(3) Remove usernames mentioned in the posts that contain the “@” symbol.

(4) Remove all topic and super-topic titles that appear in the posts, which are enclosed by the “#” symbol.

(5) Remove special markers and placeholders: Such as 【】and “d” (fold).

(6) Remove excessive line breaks, tabs, and spaces: Such as \r, \n, \t.

(7) Remove duplicate posts.

(8) Remove posts with very limited content: For example, posts that only contain a few characters.

Although automated preprocessing substantially reduced platform-specific noise, preprocessing approaches alone were insufficient to fully address highly repetitive posts and contextually irrelevant content commonly present in large-scale social media datasets. Therefore, following common practices in social media and infodemiology research, we implemented a structured multi-step data-curation procedure combining rule-based filtering and manual validation.

Frequency analysis was used to identify and exclude recurring non-study-related content, including unrelated public events (eg, “World Sleep Day”), celebrity promotion activities, fan-community discussions, commercial advertisements, astrology-related content, and other contextually irrelevant topics. Examples of excluded terms included commercial product names (eg, “ubras underwear,” “L'Oréal,” “Xiaomi 14Pro”), celebrity-related content (eg, “Xiao Zhan,” “Yang Mi”), promotional expressions (eg, “share and win a prize,” “steam eye mask,” “coupon”), astrology-related terms (eg, “horoscope,” “zodiac”), and unrelated entertainment content (eg, “full-text novels,” “tarot cards”).

To further assess contextual relevance, two trained research assistants independently reviewed subsets of the filtered posts as part of a manual validation procedure. Discrepancies were discussed until consensus was reached. Rather than relying on subjective case-by-case deletion, the filtering process followed predefined coding and exclusion rules that were consistently applied throughout the study.

The filtering procedure was conducted iteratively to refine rule consistency and reduce residual noise within the dataset. After preprocessing and validation, the final analytic dataset consisted of 106,440 posts.
